# *Pseudomonas koreensis* HLG18 improves mulberry waterlogging resilience in riparian zone by synergistically modulating endophytic microbiome and metabolic profiles

**DOI:** 10.1128/spectrum.02959-25

**Published:** 2026-02-09

**Authors:** Ting Ou, Haiying Gao, Yuping Xiong, Kun Jiang, Changyu Qiu, Kai Lin, Xiaojiao Liu, Jie Xie

**Affiliations:** 1 State Key Laboratory of Resource Insects, College of Sericulture, Textile and Biomass Sciences, Southwest University 630538, Chongqing, China; 2 Guangxi Key Laboratory of Sericultural Genetic Improvement and Efficient Breeding, Guangxi Zhuang Autonomous Region Sericultural Technology Promotion Station, Nanning, Guangxi Zhuang Autonomous Region, China; DePauw University, Greencastle, Indiana, USA

**Keywords:** waterlogging, endophyte, mulberry, microbiome, metabolites

## Abstract

Endophytes play essential roles in protecting plants against abiotic stresses. However, whether and how they enhance waterlogging resilience in mulberry through changes in host-associated microbiota and metabolites remains unclear. Here, an endophytic bacterium strain HLG18, with plant growth promotion potential, was selected and identified as *Pseudomonas koreensis* HLG18. Genome analysis revealed that it possessed multiple genes involved in phytohormone biosynthesis, mineral dissolution, and stress adaptation. Greenhouse experiments consistently indicated that *P. koreensis* HLG18 significantly stimulated mulberry growth under waterlogging stress, accompanied by enhanced antioxidant enzyme activities and osmoprotectants. Amplicon sequencing revealed distinct endospheric microbiome profiles following HLG18 treatment, with notable changes in genera, such as *Rhizorhapis*, *Bacillus*, *Caulobacter*, and *Rhodococcus*. Meanwhile, soil potassium, phosphorus, and iron levels also differed. Correlation analyses indicated that the relative abundances of *Rhizorhapis*, *Bacillus*, *Caulobacter*, and *Rhodococcus* were significantly associated with soil properties and mulberry performance. Concurrently, metabolomic profiling revealed distinct metabolic signatures between treatments, including higher levels of stress-related metabolites (e.g., L-arginine, L-isoleucine) and differences in key metabolic pathways, such as tryptophan and purine metabolism. Overall, this study uncovers that *P. koreensis* HLG18 is linked to altered microenvironmental features and host metabolic patterns under waterlogging, providing new insights into endophyte-assisted plant stress adaptation.

**IMPORTANCE:**

Waterlogging severely threatens the riparian zone of the Three Gorges Reservoir in China, causing extensive plant mortality and hindering restoration efforts. Mulberry is a promising candidate for ecological restoration, yet its growth is severely constrained under such conditions. Endophytes have emerged as key mediators of plant stress tolerance; however, their potential role in supporting mulberry adaptation to waterlogging in riparian zones remains largely unexplored. Our results show that the endophytic bacterium *Pseudomonas koreensis* HLG18 significantly promotes mulberry growth and enhances waterlogging tolerance. HLG18 inoculation is associated with distinct shifts in the host’s endophytic microbiome, soil properties, and metabolite profiles, suggesting potential links to mulberry performance under waterlogging. Our findings highlight the potential of endophytes as bioinoculants to enhance mulberry waterlogging tolerance for ecological restoration in fragile riparian ecosystems and provide a valuable reference for harnessing beneficial microbial resources in sustainable agriculture under waterlogged conditions.

## INTRODUCTION

The riparian zone constitutes a dynamic ecotone between terrestrial and aquatic ecosystems, shaped by seasonal water-level fluctuations (1). The Three Gorges Reservoir (TGR) on the Yangtze River in China is the largest water conservancy project worldwide (2). Seasonal regulation of water levels in the TGR, ranging from 145 m above sea level in summer to 175 m in winter, has created an artificial riparian zone. This reverse-seasonal waterlogging regime results in prolonged vegetation submergence, thereby causing plant mortality, soil erosion, and ecosystem degradation (3, 4). Consequently, vegetation reconstruction and reforestation have become essential management strategies in this region.

Mulberry (*Morus* L.) is a suitable tree for ecological restoration due to its rapid growth, extensive root system, and tolerance to abiotic stresses (5). Recently, mulberry has been extensively cultivated in the riparian zone to support vegetation reconstruction demands. However, frequent waterlogging and poor soil conditions have severely constrained its establishment (6–8). Our field surveys also revealed substantial variation in the growth of mulberry trees planted in 2012 in the TGR, with part of the population experiencing mortality (9). Given that mulberry development in this area is often hindered by waterlogging stress, there is an urgent need to improve its waterlogging resilience to ensure successful revegetation efforts.

Waterlogging imposes hypoxic conditions that disrupt aerobic respiration and trigger oxidative stress (10, 11), severely impairing plant development (12). Plants have evolved adaptations to mitigate these effects, such as specialized root structures and accumulation of protective compounds (13). Additionally, plant-associated microbiota, particularly endophytes, play crucial roles in stress tolerance (14–17). For example, endophytic *Bacillus*, *Pseudomonas*, and *Pantoea* spp. can promote rice seedling growth under submergence (18). Notably, our previous research revealed that mulberry performance in the TGR riparian zone is closely linked to endophytic microbial communities, suggesting their potential contribution to waterlogging tolerance (9). Nevertheless, the role of mulberry-derived endophytes in waterlogging resilience remains poorly understood.

Inoculation with exogenous microorganisms can reshape the native plant microbiota and enrich beneficial taxa that facilitate nutrient acquisition and stress adaptation (19, 20). Beyond restructuring microbial communities, exogenous inoculants can further modify soil physicochemical properties and enzyme activities, thereby creating a more favorable microenvironment for plant growth (21). These changes, in turn, interact with the host plant, influencing key metabolic pathways and modulating the synthesis of primary and secondary metabolites essential for stress tolerance and plant health (22). Therefore, microbial-assisted plant resilience involves coordinated effects on community composition, soil environment, and host metabolism.

Based on this, we hypothesize that endophytes from the riparian zone in TGR can enhance mulberry waterlogging resilience by modulating both the microbial community and host metabolic response. To test this hypothesis, we screened a beneficial endophytic bacterium and examined its effects on mulberry growth under waterlogging, alongside analyses of microbiota composition, soil properties, and metabolite profiles. This study demonstrates that improved stress resilience in mulberry parallels a concurrent shift in both the endophytic microbiome and host metabolite profile, providing new insights into endophyte-assisted plant stress adaptation.

## MATERIALS AND METHODS

### Endophytic bacteria and culture conditions

The endophytic bacteria used in this study were sourced from our laboratory’s resource library, originally isolated from mulberry trees subjected to waterlogging stress in the riparian zone of the TGR. Bacterial isolation was performed according to our established protocol (23), which involves surface sterilization of plant tissues, followed by bacterial isolation using the tissue fragmentation method. For this study, strains were activated and cultured in LB medium at 30°C with shaking at 180 revolutions per minute (rpm) for 18 h. The bacterial suspensions were centrifuged at 8,000 × *g* for 10 min, and the resulting pellets were resuspended in sterile distilled water to a final concentration of 1.0 × 10^7^ colony-forming units per milliliter (CFU/mL) for subsequent experiments.

### Plant material and growth conditions

Mulberry seeds (*Morus alba* L., Guisangyou No. 62) were surface sterilized with 75% ethanol for 3 min, followed by 5% sodium hypochlorite for 1 min, and then rinsed thoroughly with sterile distilled water. The seeds were sown in pots filled with a sterilized mixture of humus and field soil at a 4:3 (vol/vol) ratio, which had been autoclaved three times at 121°C for 30 min each. Seedlings were maintained in a growth chamber at 25°C under a 12 h photoperiod (200 µmol·m^−2^·s^−1^) and 70% relative humidity. At the two-leaf stage, seedlings were transplanted into pots (10 cm in height, 8 cm bottom diameter, and 9 cm top diameter), with three seedlings per pot. Mulberry seedlings were cultivated under consistent conditions until they reached the three-leaf stage, at which point subsequent experiments were initiated.

### Screening of plant growth-promoting endophytic bacteria

To evaluate plant growth-promoting (PGP) potential, endophytic bacteria were assessed for indole production and phosphate solubilization. For indole production, isolates were cultured in LB broth at 28°C for 3 days. Subsequently, 500 µL of the culture supernatant was mixed with 1 mL Salkowski reagent (prepared with 50 mL of 3.5 M perchloric acid and 1 mL of 0.5 M FeCl_3_ solution) and incubated in darkness for 30 min. Development of a pink-red color indicated indole production. Phosphate solubilization was tested by inoculating isolates onto Pikovskaya’s agar plates and incubating at 28°C for 3 days. Clear zones around colonies indicated positive activity.

Based on these PGP traits, strain HLG18 was selected for further analysis. Time-course quantification of indole production and phosphate solubilization by HLG18 was performed following Suzuki et al. (24) and Dame et al. (25), respectively.

### Identification of the HLG18 strain

The morphological characteristics of strain HLG18 were recorded after incubation on LB medium at 28°C for 48 h. Cellular morphology was further examined using a scanning electron microscope (Hitachi SU3500, Japan). Gram staining was performed and visualized under an optical microscope following Becerra et al. (26). Biochemical tests were conducted using the HK-MID-66 identification kit (HUANKAI, China) according to the manufacturer’s instructions. Genomic DNA was extracted using a PrepMan Ultra Sample Preparation Reagent kit (Applied Biosystems, Palo Alto, CA, United States). The 16S ribosomal RNA (rRNA) gene was amplified using primers 27F and 1492R. A phylogenetic tree was constructed using the neighbor-joining method in MEGA X (version 10.1.8) with 1,000 bootstrap replicates (27).

### Whole-genome sequencing of strain HLG18

Genomic DNA from strain HLG18 was extracted using the QIAamp DNA mini kit (Qiagen, Germany) following the manufacturer’s instructions. Whole-genome sequencing was performed on the PromethION platform. After quality control, the sequencing data were assembled using Flye software (version 2.7). Gene prediction was carried out with Prodigal (version 2.6.3) to identify protein-coding sequences. Transfer RNA (tRNA), rRNA, and non-coding RNA genes were predicted using tRNAscan-SE (version 2.0), RNAmmer (version 1.2), and Infernal (version 1.1.3), respectively. Functional annotation was conducted using TIGRFAMs, Pfam, and the Clusters of Orthologous Groups protein database. A circular chromosome map was generated with Circos (version 0.69). Biosynthetic gene clusters for secondary metabolite production were predicted using antiSMASH (version 4.0).

### Evaluation of beneficial potential of HLG18

The 1-aminocyclopropane-1-carboxylic acid (ACC) deaminase activity was assessed using Dworkin and Foster (DF) medium and ACC-supplemented DF (ADF) medium. Strain HLG18 was cultured in DF medium, then transferred to ADF medium (3 mM ACC) and incubated at 28°C with shaking at 180 rpm for 2 days. Growth in ADF medium indicated ACC utilization as a sole nitrogen source. Siderophore production was evaluated on chrome azurol S agar (28), where orange halos around colonies indicated positive activity. Potassium solubilization was assessed on modified Aleksandrov agar supplemented with potassium feldspar powder after incubation at 28°C for 3 days (29, 30). Cellulase activity was determined on carboxymethyl cellulose agar (31). Nitrogen-fixing ability was assessed using Ashby agar according to Liu et al. (32).

### Co-cultivation of HLG18 and mulberry to assess plant growth promotion

Mulberry seedlings were inoculated with 30 mL of bacterial suspension at 3-day intervals (three applications total). Seedlings receiving sterile water served as the control. Each treatment was replicated 20 times. Fourteen days after the final inoculation, eight randomly selected seedlings per treatment were measured for the length and biomass of roots and stems. Root architecture was analyzed using the GXYB Root Analysis System (GXY-A, Tpyn Agriculture Technology Co., Ltd., Hangzhou, China).

### Assessment of HLG18 effects on mulberry waterlogging tolerance

HLG18-inoculated seedlings (grown for 60 days) were exposed to root waterlogging (RW; water maintained 5 cm above the soil surface) or complete waterlogging (CW; water maintained 40 cm above the soil surface). Seedlings treated with sterile water served as the control group. After 20 days of waterlogging, morphological parameters, including fresh weight of shoot and root, root length, and ground diameter, were measured. Concurrently, mulberry leaves were harvested and flash-frozen in liquid nitrogen for physiological analysis. Following the waterlogging treatment, seedlings were returned to normal growth conditions, with 30 mL sterile water added every 3 days. After a 31-day recovery period, root length, as well as fresh and dry weights of roots and stems from 10 randomly selected seedlings, were measured to evaluate recovery potential.

### Physiological and histochemical assays

Soluble sugar (SS), soluble protein (SP), and malondialdehyde (MDA) contents, and the activities of antioxidant enzymes, including superoxide dismutase (SOD) and peroxidase (POD), were quantified in mulberry leaves using commercial biochemical kits (Suzhou Comin Biotechnology Co., Ltd., China, product numbers KT-1-Y, KMSP-1-W, MDA-1-Y, SOD-1-W, POD-1-W) according to the manufacturer’s instructions.

*In situ* cell viability and root integrity were assessed via Evans blue staining. Tissues were immersed in 2.5 mg/mL Evans blue (pH 7.0; Dingguo Changsheng Biotechnology Co., Ltd., Beijing, China) for 30 min, followed by thorough rinsing and examination under an optical microscope (Leica, Germany).

### Detection of mulberry-associated bacterial communities

To investigate the impact of HLG18 inoculation on bacterial community composition, rhizosphere soil, root, and stem samples were collected 14 days after inoculation. Twelve seedlings were randomly selected, with three pooled per biological replicate. Rhizosphere soil within approximately 1 mm of the root surface was carefully collected with a sterile scalpel. Roots were retrieved with sterile tweezers, gently rinsed to remove debris, and surface-sterilized with 75% ethanol for 5 min, followed by flame sterilization. Stem segments approximately 5  cm above the soil surface were harvested similarly. All samples were stored at −80°C for subsequent processing. Seedlings treated with sterile water, as used in a previous study (23), served as the control group and were sampled simultaneously with this experimental seedling for comparative analysis in the current study.

DNA extraction and 16S rRNA gene sequencing followed previously described methods (23). Briefly, total DNA was extracted using the FastDNA Spin Kit (MP Biomedicals, Santa Ana, CA, USA). DNA concentration was measured with a NanoDrop 2000 spectrophotometer (Thermo Scientific, Wilmington, USA). The V3–V4 hypervariable region was amplified with primers 338F (5′-ACTCCTACGGGAGGCAGCA-3′) and 806R (5′-GGACTACHVGGGTWTCTAAT-3′).

Sequencing was performed on an Illumina MiSeq platform (Illumina, San Diego, USA) by Majorbio Bio-Pharm Technology Co., Ltd. (Shanghai, China). Raw reads were quality-filtered using FASTP (version 0.20.0) and processed with USEARCH (version 7.0). Operational taxonomic units (OTUs) were clustered at 97% similarity using UPARSE (version 11.0), and OTUs assigned to mitochondria or chloroplasts were removed. Taxonomy was performed using the Ribosomal Database Project Classifier against the SILVA (version 138) at 70% confidence threshold. α-Diversity indices were calculated in Mothur. Diversity differences among groups were evaluated by one-way analysis of variance (ANOVA). β-Diversity was analyzed using non-metric multidimensional scaling (NMDS) based on Bray-Curtis dissimilarity at the genus level. Venn diagrams were generated with the VennDiagram package in R. Differences in genera-level abundances were tested by the Wilcoxon rank-sum test. Spearman correlation coefficients between bacterial genera, environmental variables, and mulberry growth parameters (Table S1) were visualized as a heat map.

### Soil physicochemical analysis

To evaluate the effects of HLG18 inoculation on soil properties, approximately 40 g of soil was collected per pot after 14 days of treatment. Each experimental group included four biological replicates. Soil samples were oven-dried at 105°C and passed through a 2 mm mesh sieve prior to analysis. Subsequently, key physicochemical parameters (organic carbon, organic matter, available phosphorus, total potassium, available potassium, and available iron) were determined using standardized methods according to the Chinese Agricultural Soil Testing Protocols (23, 33, 34).

### Root metabolites analysis

To investigate the influence of strain HLG18 on root metabolism, a total of 18 seedlings were randomly harvested 14 days after inoculation. Six biological replicates were prepared, each consisting of three pooled seedlings. Seedlings treated with sterile water, as used in a previous study (23), served as the control group and were sampled simultaneously with this experimental seedling for comparative analysis in the current study. Approximately 0.5 g of root tissue was homogenized in 400 µL of 80% methanol (containing 2 mg/L L-2-chlorophenylalanine) using a pre-cooled tissue grinder. Chloroform was added during grinding at 50 Hz for 6 min. Extracts were sonicated, centrifuged (13,000 × *g*, 15 min, 4°C), and transferred to vials for ultra-high-performance liquid chromatography coupled with tandem mass spectrometry (UHPLC-MS/MS) analysis. A pooled quality control sample, prepared from 20 µL aliquots of each extract, was used to monitor analytical stability.

Metabolite separation was performed on a Thermo UHPLC system with an Acquity BEH C18 column. Mass spectrometric data were acquired using a Thermo Q Exactive mass spectrometer with an electrospray ionization source in both positive and negative ion modes. Data processing and statistical analysis were conducted via the online platform provided by Majorbio Biotech Co., Ltd. (Shanghai, China). Orthogonal partial least squares discriminant analysis (OPLS-DA) was used to compare metabolic profiles between treatments. Metabolites with a variable importance in projection (VIP) >1 and *P* <0.05 were considered significantly different. Differential metabolites were functionally annotated using the KEGG database. Pearson correlations between root metabolites and bacterial genera were calculated and visualized as a heatmap.

### Statistical analysis

All statistical analyses were performed using GraphPad Prism 8.0. Group differences were evaluated by two-tailed unpaired Student’s *t*-tests or one-way ANOVA, followed by Tukey’s or Dunnett’s post hoc tests as appropriate. Data are presented as the mean ± standard error of the mean (SEM). *P* values <0.05 were considered statistically significant.

## RESULTS

### Screening of plant growth-promoting endophytic bacteria and identification of strain HLG18

Endophytic isolates from vigorously growing mulberry trees in the TGR riparian zone were screened for phosphate solubilization and indole production. A total of six isolates exhibited both capabilities (Table S2). Notably, strain HLG18 exhibited the highest activity compared to other strains and was selected for further study. When cultivated in LB medium supplemented with tryptophan, HLG18 secreted significantly higher levels of indole compared with LB medium alone, reaching a peak concentration of 22.53 mg/mL on day 9 (Fig. S1A). In parallel, HLG18 showed efficient phosphate solubilization in Pikovskaya’s medium, with soluble phosphate concentrations reaching 38.01, 62.55, and 21.74 μg/mL on days 3, 4, and 5, respectively (Fig. S1B).

**Fig 1:**
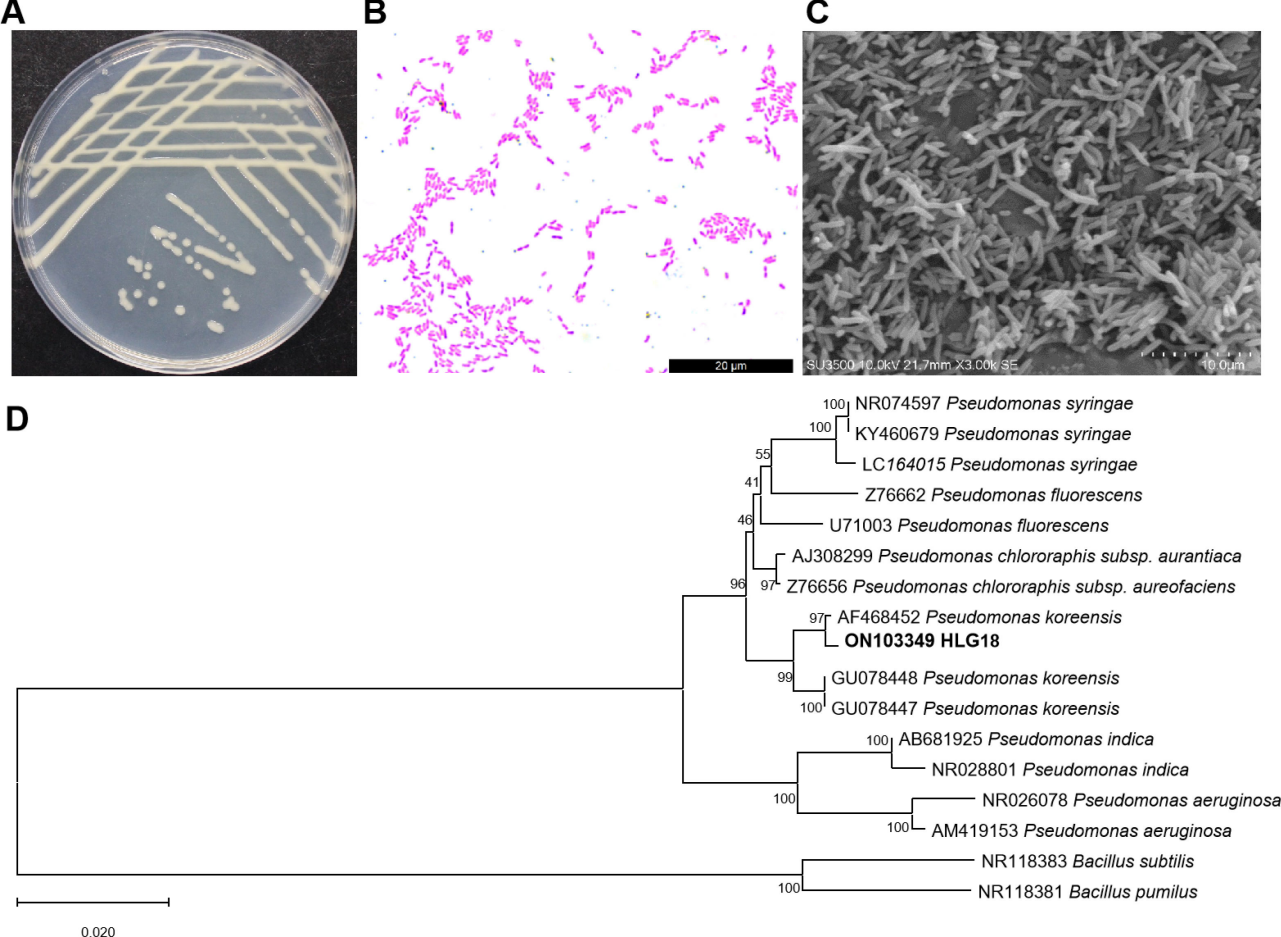
Identification of *P. koreensis* HLG18. (**A**) Colony morphology of HLG18 grown on LB agar after 48 h of incubation at 28°C. (**B**) Gram staining of HLG18 was observed under a light microscope. (**C**) Scanning electron microscopy showing the cellular morphology of HLG18. (**D**) Phylogenetic tree based on 16S rRNA gene sequences, constructed using the neighbor-joining method in MEGA-X version 10.1.8 with 1,000 bootstrap replicates.

Colony morphology observations revealed that strain HLG18 formed smooth, white colonies on LB agar after 48 h of incubation at 28°C (Fig. 1A). Gram staining and scanning electron microscopy analyses indicated that it is a Gram-negative, rod-shaped bacterium with approximately 0.8 μm in diameter and 5 μm in length (Fig. 1B and C). Biochemical characterization showed that HLG18 was negative for nitrate reduction and peptone hydrolysis but positive for sucrose and citrate utilization (Table S3). Phylogenetic analysis based on the full-length 16S rRNA gene sequence of HLG18 indicated that it is most closely related to *Pseudomonas koreensis* (Fig. 1D). Based on its morphological, biochemical, and molecular characteristics, the isolate was identified as *P. koreensis* HLG18.

### Genomic analysis of *P. koreensis* HLG18

The complete genome of *P. koreensis* HLG18 consists of a 6.8 Mb circular chromosome with a GC content of 60.43% and no endogenous plasmids, containing 5,644 predicted coding sequences (CDS) (Fig. S2; Table S4). Comparative genomic analysis revealed that HLG18 possesses a larger genome and a higher CDS count than other *P. koreensis* strains, although other features remained conserved. AntiSMASH analysis predicted eight putative biosynthetic gene clusters, which comprised four non-ribosomal peptide synthetases, along with clusters associated with ranthipeptide, N-acetylglutaminylglutamine amide, redox-cofactor, and arylpolyene biosynthesis (Table S5), indicating the potential to produce diverse bioactive compounds.

Genome annotation revealed a wide array of functional genes related to plant growth promotion and stress-tolerance enhancement (Table 1). These include genes involved in phosphate solubilization (e.g., *pitA*, *ppk*, *ppx*) (14) and indole-3-acetic acid synthesis (e.g., *iaaM*, *iacA*) (35, 36). In addition, genes encoding proteins for nitrogen fixation (*nifH*) (37), siderophore biosynthesis (*fur*, *pch*) (38), ACC deaminase (*acd*) (39), cellulase (*celA*) (40), and potassium solubilization (*kdpD*) (41) were identified. These traits are correlated with improved nutrient acquisition, growth, and stress adaptation. Correspondingly, functional assays confirmed that *P. koreensis* HLG18 produces ACC deaminase, siderophores, and cellulase and is capable of nitrogen fixation and potassium solubilization (Fig. S1C through G).

**TABLE 1:** Potentially beneficial genes of *P. koreensis* HLG18 strain for plant growth

Position		Gene	Gene annotation	Description
From	To			
278,276	279,751	*pitA*	Inorganic phosphate transporter	Phosphate solubilization
405,072	405,896	*ppk2*	Polyphosphate kinase 2	
4,889,447	4,890,949	*ppx*	Exopolyphosphatase	
4,891,016	4,893,241	*ppk1*	Polyphosphate kinase 1	
4,554,771	4,556,453	*iaaM*	Tryptophan 2-monooxygenase	IAA synthesis
5,994,671	5,995,828	*iacA*	Indole-3-acetate monooxygenase	
3,953,079	3,953,465	*nifU*	Nitrogen fixation protein	Nitrogen fixation
5,830,234	5,831,361	*kdpD*	K^+^-sensing histidine kinase	Potassium solubilization
2,895,048	2,895,956	*acd*	1-Aminocyclopropane-1-carboxylate deaminase	ACC deaminase
5,369,181	5,370,536	*bcsA*	Catalytic subunit of cellulose synthase	Cellulase
5,700,215	5,701,111	*bcsL*	Acetyltransferase involved in cellulose biosynthesis	
317,444	319,735	*celA1*	Cellulase	
1,868,388	1,868,711	*fur*	Fe^2+^ or Zn^2+^ uptake regulation protein	Siderophores synthesis
647,008	647,832	*pch*	Iron transporter	
1,174,484	1,175,791	*rfbX*	Oligosaccharide flippase family protein	Polysaccharides synthesis
1,181,355	1,182,947	*gumC*	Lipopolysaccharide biosynthesis protein	
3,317,975	3,319,969	*flaA1*	Polysaccharide biosynthesis protein	
399,795	400,355	*gcvR*	Glycine cleavage system protein R	Glycine betaine synthesis
663,423	664,274	*proX*	Glycine betaine ABC transporter substrate-binding protein	
3,693,904	3,694,287	*gcvH*	Glycine cleavage system protein GcvH	
3,698,840	3,699,964	*gcvT*	Glycine cleavage system aminomethyltransferase GcvT	
4,851,989	4,854,862	*gcvP*	Glycine dehydrogenase (aminomethyl-transferring)	
795,629	796,504	*speE*	Polyamine aminopropyltransferase	Polyamine synthesis
1,274,037	1,275,134	*potD*	Extracellular solute-binding protein	
1,275,204	1,276,082	*potC*	ABC transporter permease subunit	
1,276,079	1,276,993	*potB*	ABC transporter permease subunit	
1,276,990	1,278,132	*potA*	Polyamine ABC transporter ATP-binding protein	
3,269,982	3,270,737	*speE*	Spermidine synthase	
3,905,901	3,906,677	*viuB*	Siderophore-interacting protein	Siderophore metabolic process
4,099,802	4,100,827	*hemH*	Ferrochelatase	
1,281,109	1,282,599	*adhE*	Aldehyde dehydrogenase	Oxidation-reduction process
1,465,125	1,466,558	*katE*	Catalase	
3,787,855	3,788,451	*sodA*	Superoxide dismutase	
4,840,894	4,841,532	*ahpC*	Peroxiredoxin	
1,068,194	1,068,406	*cspC*	Cold-shock protein	
3,957,187	3,958,005	*suhB*	Inositol monophosphatase	Inositol
1,661,350	1,662,780	*ansP*	GABA permease	γ-aminobutyric acid
5,511,340	5,512,731	*gabP*	GABA permease	

Beyond nutrient-related traits, the genome of *P. koreensis* HLG18 carries genes involved in stress-responsive pathways, including those for glycine-betaine, polyamine, inositol, and γ-aminobutyric acid metabolisms, which are known to mitigate oxidative stress and enhance plant stress tolerance. Meanwhile, genes encoding cold-shock proteins and oxidative stress enzymes, such as superoxide dismutase (*sodA*) and catalase (*katE*), were also identified (42). These features suggest that *P. koreensis* HLG18 possesses intrinsic potentials for survival under harsh environments and may contribute to stress resilience in its host plant.

### Growth-promoting effect of *P. koreensis* HLG18 on mulberry

**Fig 2:**
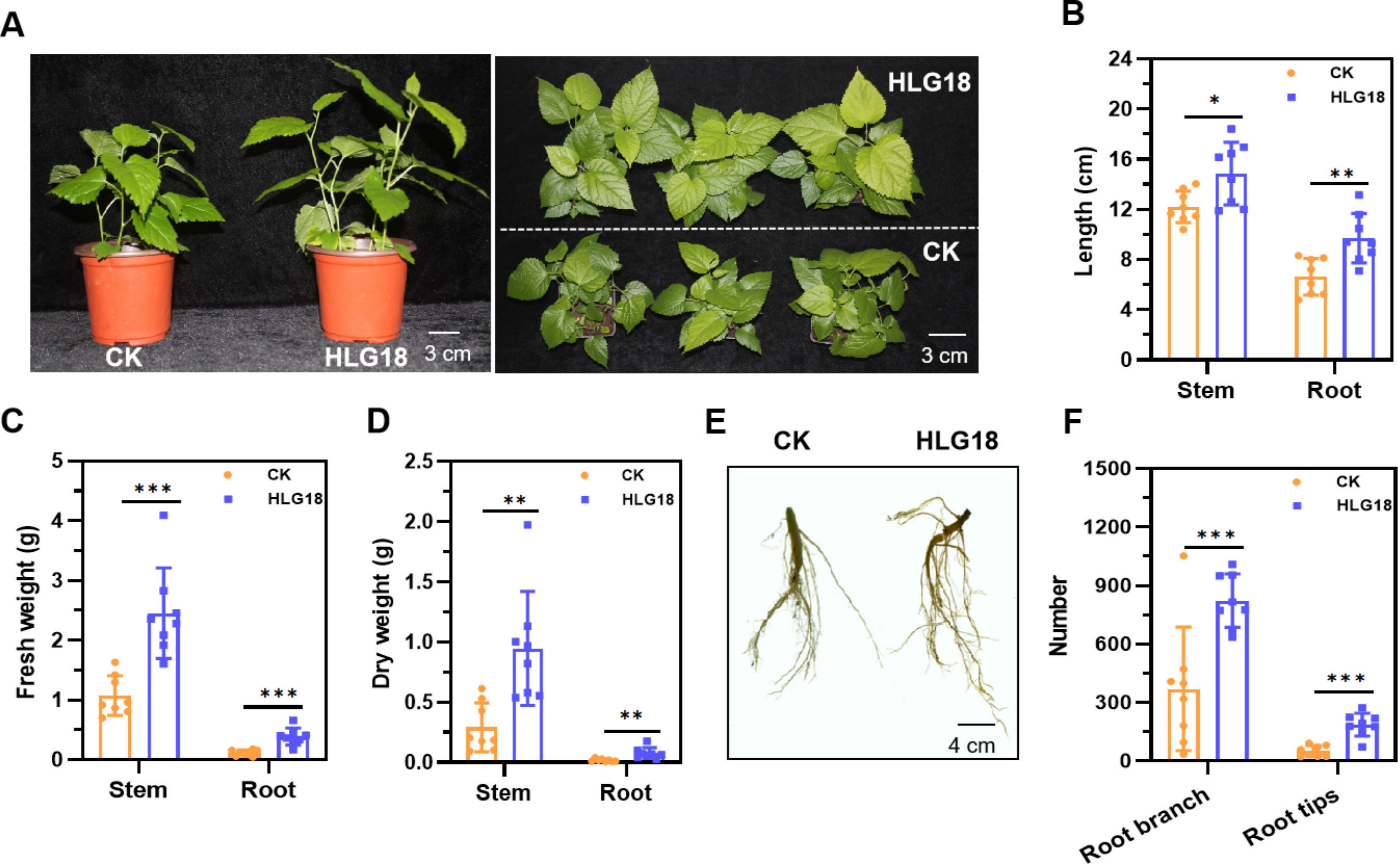
Growth-promoting effects of *P. koreensis* HLG18 on mulberry seedlings. (**A**) Representative images of mulberry seedlings 14 days after inoculation with *P. koreensis* HLG18 or water control (CK). Left: side view; right: top view. (**B–D**) Quantitative measurements of shoot and root traits: length (**B**), fresh weight (**C**), and dry weight (**D**). (**E**) The representative photograph of mulberry root architectures. (**F**) Quantification of root branches and tip numbers. Mulberry seedlings at the four-leaf stage were inoculated via root irrigation with 30 mL of bacterial suspension or sterile water and applied three times at 3-day intervals. Plants were harvested after 14 days for phenotypic and morphological assessment. Data are presented as mean ± SEM (*n* = 8). Statistical significance was determined by the one-way ANOVA with Tukey’s tests, *P < 0.05, **P < 0.01, ***P < 0.001.

Given that *P. koreensis* HLG18 harbors multiple genes associated with the biosynthesis of potential PGP compounds, its effects on mulberry growth were further evaluated. Co-cultivation assays indicated that *P. koreensis* HLG18 significantly promoted seedling growth (Fig. 2A). Specifically, mulberry inoculated with *P. koreensis* HLG18 exhibited a 21.6% increase in stem length and a 46.3% increase in root length compared to the control (Fig. 2B). Biomass accumulation was also improved, with both fresh and dry weights of stems and roots significantly higher (*P* < 0.01) in HLG18-treated seedlings (Fig. 2C and D).

Notably, *P. koreensis* HLG18 also promoted root architecture (Fig. 2E). Inoculated plants developed a denser and more highly branched root network, with the number of root tips and lateral branches increasing by 267.3% and 122.2%, respectively, compared with uninoculated plants (Fig. 2F). Together, these results demonstrate that *P. koreensis* HLG18 promotes mulberry growth by stimulating both shoot and root development, indicating its potential as an effective bioinoculant for improving plant productivity.

### *P. koreensis* HLG18 improves mulberry tolerance to waterlogging stress

**Fig 3:**
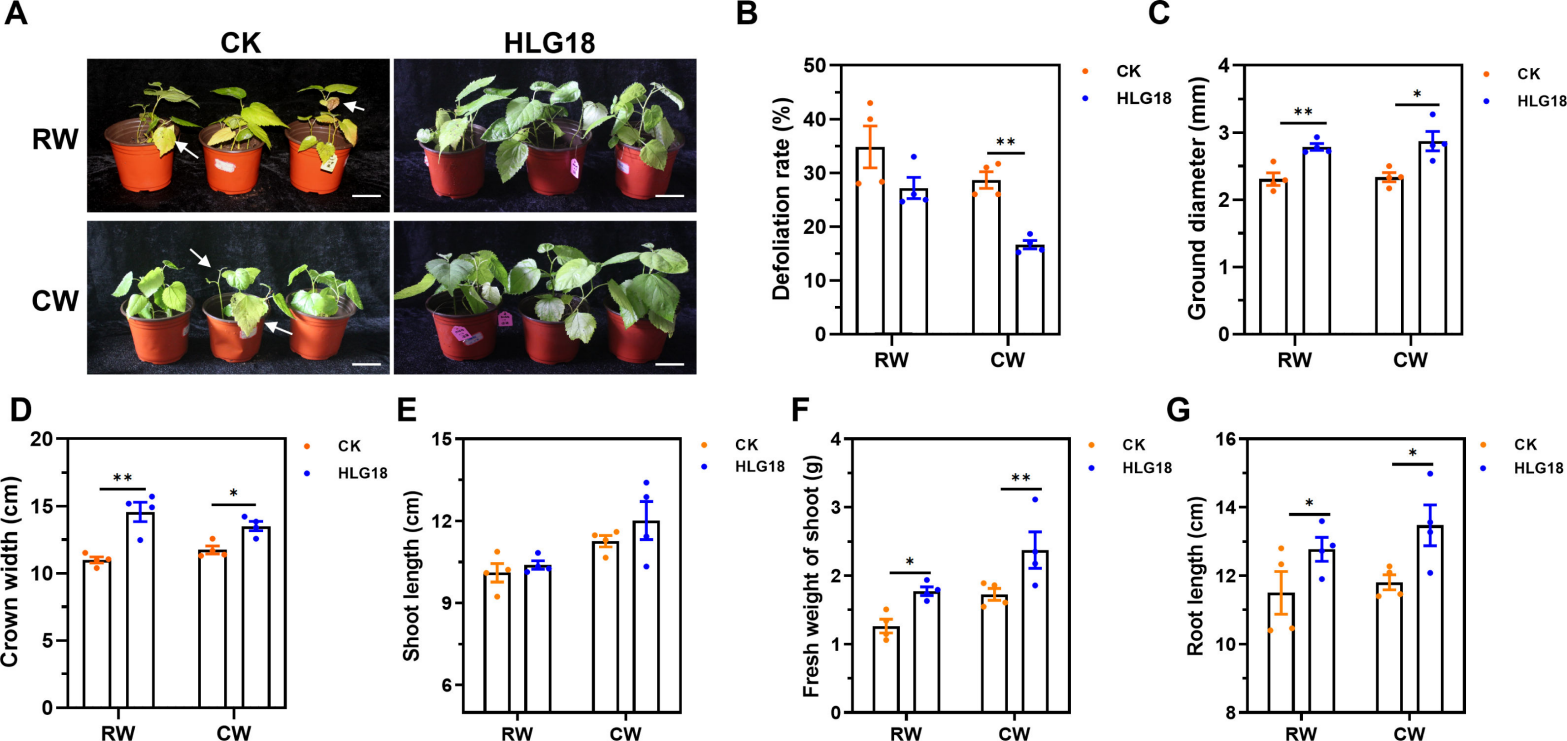
Inoculation with *P. koreensis* HLG18 enhances mulberry tolerance to waterlogging stress. (**A**) Representative images of mulberry seedlings inoculated with *P. koreensis* HLG18 or water control (CK) under RW and CW conditions. Arrows indicate symptomatic leaves exhibiting chlorosis or water-soaked lesions. Scale bars = 5 cm. (**B–G**) Quantitative assessment of mulberry growth perimeters, including defoliation rate (**B**), ground diameter (**C**), crown width (**D**), shoot length (**E**), shoot fresh weight (**F**), and root length (**G**). Seedlings were pretreated by irrigation with *P. koreensis* HLG18 or water for 60 days, followed by waterlogging treatment until visible stress symptoms appeared. Data were collected after 20 days of waterlogging. Values represent mean ± SEM (*n =* 4). A significant difference was determined by the one-way ANOVA with Tukey’s tests, *P < 0.05, **P < 0.01. RW, root waterlogging; CW, complete waterlogging.

To evaluate whether *P. koreensis* HLG18 enhances mulberry tolerance to waterlogging, inoculated seedlings were subjected to either root waterlogging (RW) or complete waterlogging (CW). After 2 weeks, inoculated plants exhibited more vigorous growth than non-inoculated controls (Fig. 3A). *P. koreensis* HLG18 treatment significantly reduced defoliation (*P* < 0.01), decreasing it by 42% under CW conditions (Fig. 3B). Inoculated plants also displayed increased ground diameter (RW: 20.7%; CW: 23%) and crown width (RW: 32.6%; CW: 15.1%) (Fig. 3C and D). Moreover, stem and root biomass and length were significantly greater in treated seedlings under both waterlogging conditions (Fig. 3E through G).

**Fig 4:**
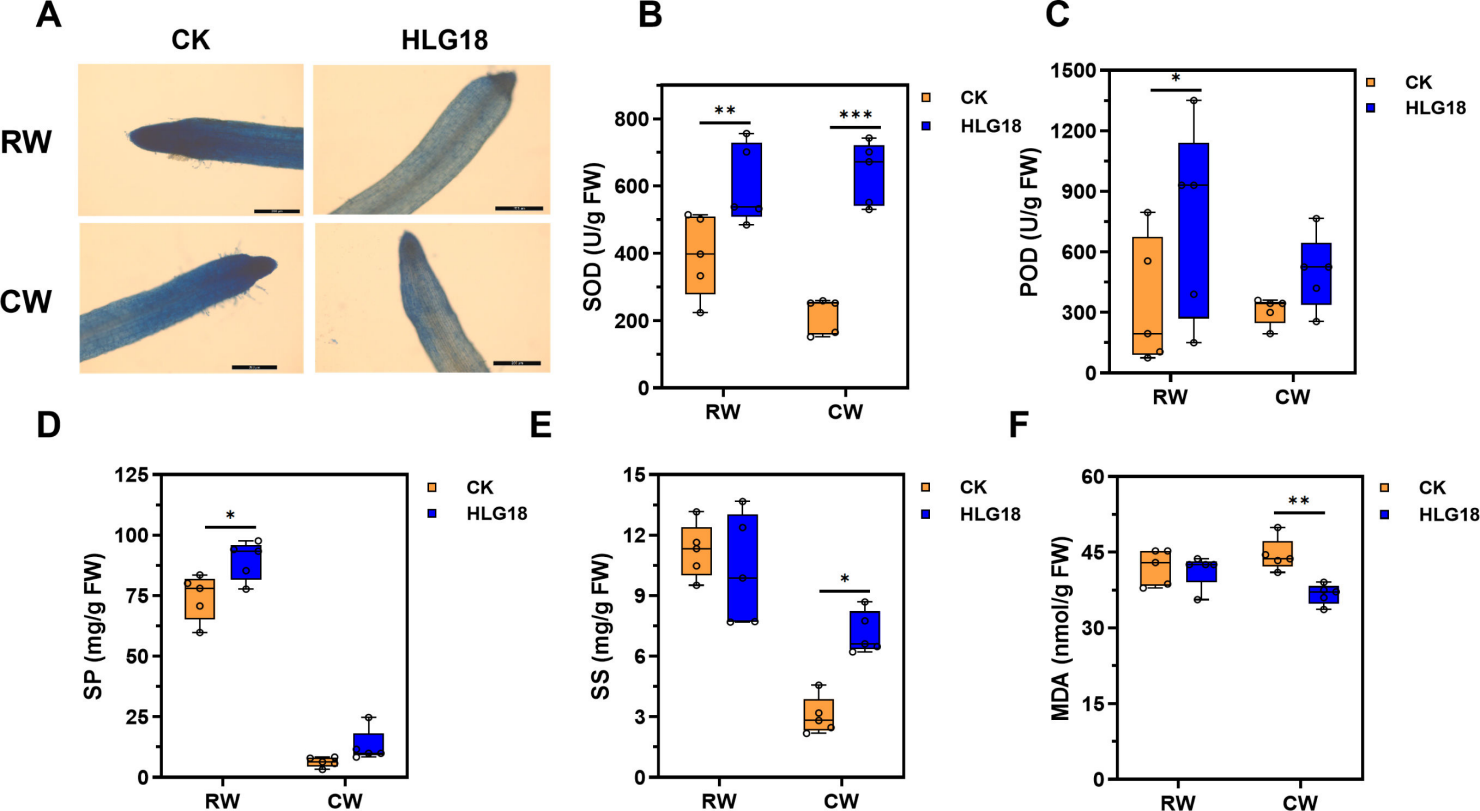
*P*. *koreensis* HLG18 mitigates waterlogging-induced cellular injury and enhances stress-related metabolite accumulation in mulberry. (**A**) Visualization of cell death in mulberry roots following 20 days of waterlogging stress, as assessed by Evans blue staining. Scale bars = 100 µm. (**B–F**) Physiological and biochemical responses measured in mulberry leaves: (**B**) SOD activity, (**C**) POD activity, (**D**) SP content, (**E**) SS content, and (**F**) MDA content. Data are shown as mean ± SEM (*n =* 5). A significant difference was determined by the one-way ANOVA with Tukey’s tests, *P < 0.05, **P < 0.01, ***P < 0.001. RW, root waterlogging; CW, complete waterlogging.

Physiological analyses indicated that waterlogging-induced cell death increased with stress severity, whereas HLG18 markedly alleviated this damage, as evidenced by Evans blue staining (Fig. 4A). Consistent with reduced injury, antioxidant enzyme activities were elevated in inoculated plants. Specifically, *P. koreensis* HLG18 significantly (*P* < 0.001) facilitated SOD activity, which increased by 52.9% (RW) and 195.7% (CW) (Fig. 4B). POD activity was also enhanced, rising by 117.4% under RW (Fig. 4C). In parallel, soluble protein content increased by 20.4% (RW) (Fig. 4D), and soluble sugar content rose by 134.9% (CW) (Fig. 4E). Conversely, MDA content, a marker of lipid peroxidation, decreased by 17.6% under CW in *P. koreensis* HLG18-treated plants (Fig. 4F), indicating reduced oxidative membrane damage.

To evaluate recovery after stress, seedlings were returned to normal conditions for 31 days following waterlogging. Inoculated plants showed higher vigor and produced more new leaves than controls (Fig. S3A). Stem biomass increased by 82.7% (RW) and 44.0% (CW), and ground diameter improved by 38.2% and 23.9%, respectively (Fig. S3B and C). Meanwhile, root fresh weight increased by 254.6% (RW) and 107.0% (CW) compared to controls (Fig. S3D and E). Root length was also significantly higher (*P* < 0.001), increasing by 44.7% (RW) and 19.5% (CW) (Fig. S3F). Collectively, these results demonstrate that *P. koreensis* HLG18 markedly improves mulberry waterlogging tolerance by stimulating growth, strengthening antioxidant capacity, mitigating oxidative damage, and accelerating post-stress recovery.

### *P. koreensis* HLG18 contributed to variation in the mulberry endophytic microbiome

To determine whether exogenous inoculation with *P. koreensis* HLG18 influences the mulberry-associated microbiome, 16S rRNA amplicon sequencing was performed on rhizosphere soil, stem, and root samples from inoculated and control plants. A total of 1,310,311 high-quality reads were generated, clustering into 1,660 unique OTUs. Shannon-based rarefaction curves reached saturation across samples (Fig. S4), indicating sufficient sequencing depth. OTU richness exhibited a decline from rhizosphere to endosphere compartments (Fig. S5A), and α-diversity (Ace and Shannon indices) was consistently lower in endophytic tissues than in the rhizosphere (Fig. S5B).

The β-diversity analysis using NMDS revealed clear separation between control and HLG18-treated communities in both the rhizosphere and endosphere compartments (Fig. S6A). Taxonomic profiling showed that rhizosphere communities were largely stable, whereas observable compositional shifts occurred in the endophytic compartments (Fig. S6B). At the order level, the root endosphere showed decreased relative abundances of Rhizobiales (CK: 34.57%; HLG18: 17.45%) and Flavobacteriales (CK: 17.53%; HLG18: 31.07%), while Bacillales (CK: 0%; HLG18: 0.54%) and Sphingomonadales (CK: 17.53%; HLG18: 31.07%) increased following inoculation. In stems, Rhizobiales (CK: 11.31%; HLG18: 39.69%) and Sphingomonadales (CK: 4.30%; HLG18: 13.07%) increased, whereas Burkholderiales declined (CK: 27.83%; HLG18: 3.80%). At the genus level, rhizosphere assemblages remained broadly similar between treatments, but endophytic communities were not significantly different (Fig. S6B). *Bacillus* (CK: 0.13%; HLG18: 2.15%), *Rhizorhapis* (CK: 16.66%; HLG18: 29.65%), and *Cellvibrio* (CK: 1.27%; HLG18: 4.88%) were enriched in roots, while *Devosia* (CK: 0.68%; HLG18: 4.26%), *Sphingomonas* (CK: 4.07%; HLG18: 12.61%), and *Methylorubrum* (CK: 3.62%; HLG18: 20.97%) were enriched in stems.

**Fig 5:**
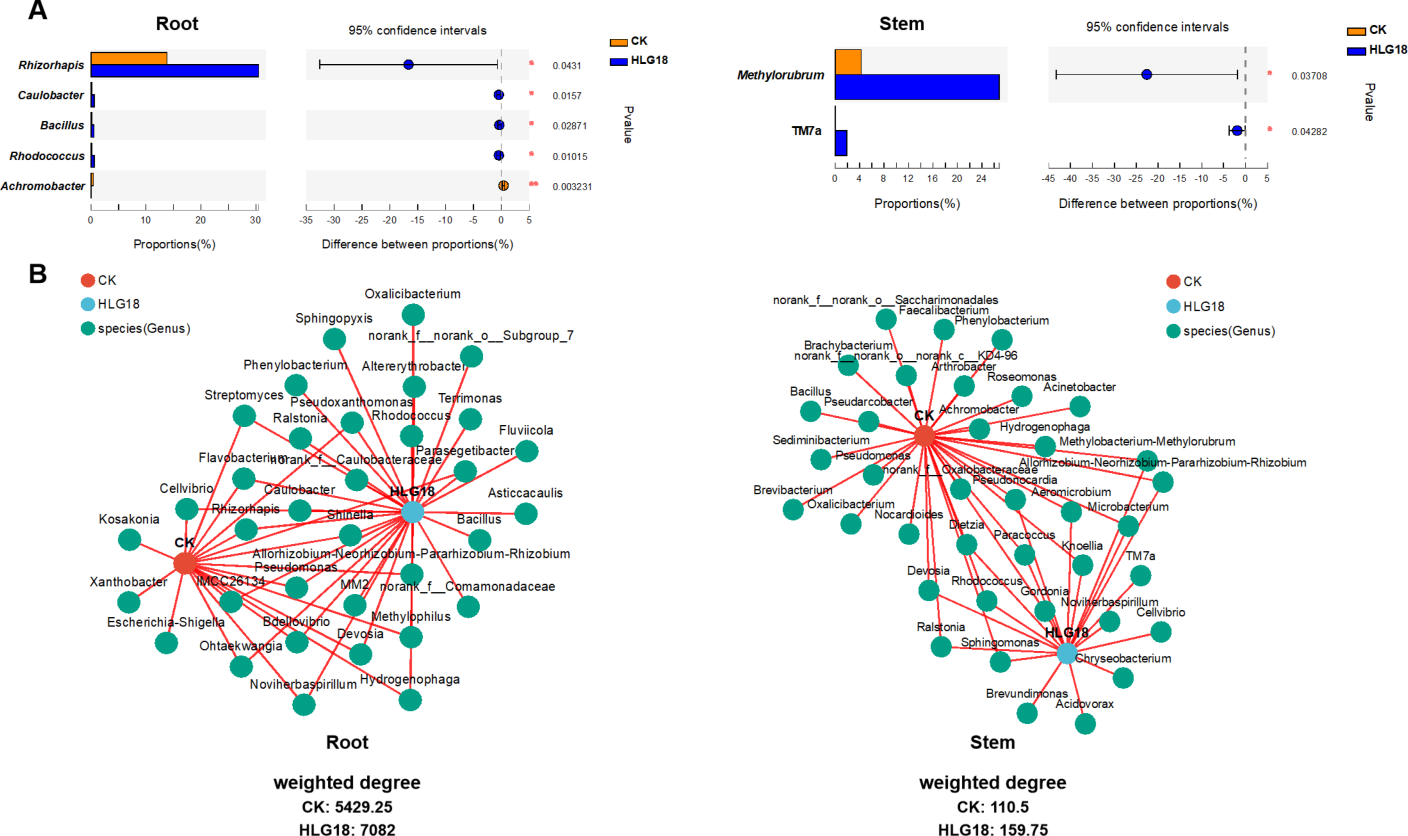
*P*. *koreensis* HLG18 selectively modulates endophytic bacterial genera in mulberry. (**A**) Comparison of the relative abundances of the top 10 endophytic bacterial genera (based on total mean abundance) in mulberry roots (left) and stems (right). Genera showing statistically significant differences between treatments are marked. Significant difference was determined by two-tailed Student’s *t*-test: *P < 0.05, **P < 0.01. (**B**) Co-occurrence network analysis of endophytic bacterial genera in mulberry roots (left) and stems (right). Nodes represent bacterial genera or sample types, with node size proportional to relative sequence abundance. Edges connect sample and genus nodes, indicating co-occurrence relationships. Only genera with total sequence counts >50 are shown. The weighted degree of a node represents the total number of sequence-based associations connected to that node.

To characterize community interaction patterns, co-occurrence network analysis was performed. Compared with the controls, *P. koreensis* HLG18-inoculated groups exhibited more complex and modular networks (Fig. 5B; Fig. S7), with more networked genera and higher weighted degree (roots: 7,082 vs 5,429.25; stems: 159.75 vs 110.50) (Fig. 5B). Inoculated communities also showed a higher average node degree (34.63) compared with controls (22.47), indicating increased connectivity and potentially stronger cooperative interactions (Fig. S7). These results suggest that *P. koreensis* HLG18 is associated with a modified endophytic community structure and enhanced network complexity, particularly in mulberry roots and stems.

### Shifts in endophytic core genera following *P. koreensis* HLG18 correlate with soil property and mulberry growth

**Fig 6:**
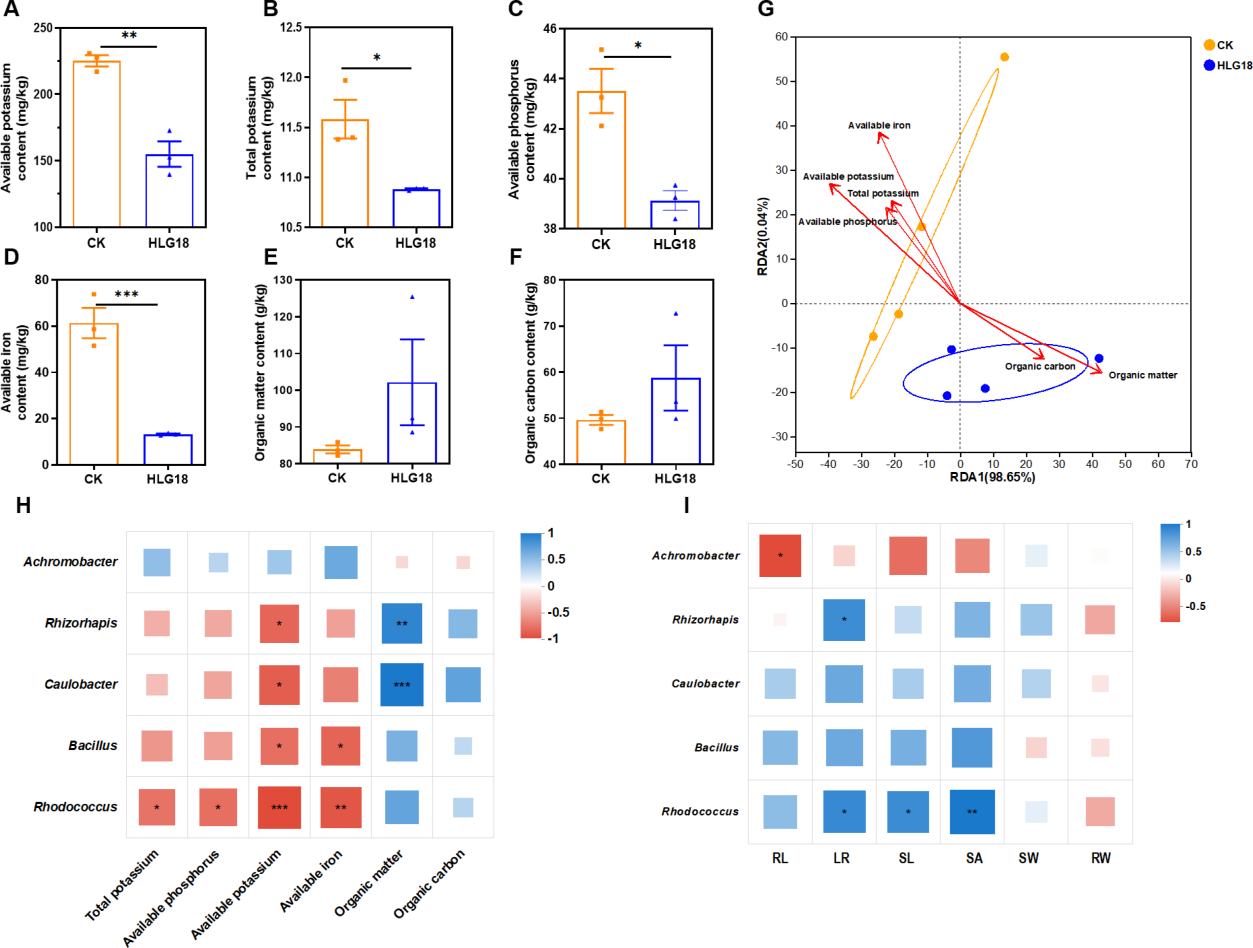
Increased core endophytic taxa following *P. koreensis* HLG18 inoculation exhibit strong interactions with soil physicochemical traits and mulberry growth performance. (**A–F**) Effects of *P. koreensis* HLG18 inoculation on soil properties, including available potassium (**A**), total potassium (**B**), available phosphorus (**C**), available iron (**D**), organic matter (**E**), and organic carbon content (**F**). Data are shown as mean ± SEM (*n =* 3). Significant difference was determined by the Student’s *t*-test, *P < 0.05, **P < 0.01, ***P < 0.001. (**G**) RDA illustrating correlations between soil physicochemical parameters and the top 30 root endophytic genera. Arrow length indicates explanatory power of each factor, and angle between arrows indicates correlation type (acute = positive, obtuse = negative). (**H**) Spearman correlation heatmap between soil properties and the core enriched endophytic genera. (**I**) Spearman correlation heatmap between mulberry growth traits and the core enriched endophytic genera. Growth traits include shoot length (SL), main root length (RL), shoot weight (SW), root weight (RW), root tip number (LR), and root surface area (SA). Color intensity indicates correlation strength (*r*-value). *P < 0.05, **P < 0.01, ***P < 0.001.

The key endophytic taxa were greatly altered following inoculation with *P. koreensis* HLG18. Specifically, *Rhizorhapis*, *Bacillus*, *Caulobacter*, and *Rhodococcus* were markedly increased in roots after HLG18 treatment, whereas *Achromobacter* significantly declined (*P* < 0.05) (Fig. 5A). In stems, *Methylorubrum* and *TM7a* were also significantly enriched (*P* < 0.05). Concurrently, soil property analyses indicated that application of *P. koreensis* HLG18 was accompanied by significant decreases in soil potassium, phosphorus, and iron levels (Fig. 6A through D), alongside an increase in organic matter and carbon contents (Fig. 6E and F). Redundancy analysis (RDA) further showed that *P. koreensis* HLG18 treatment was positively correlated with organic matter and carbon, but negatively associated with the other nutrient variables measured (Fig. 6G).

To link the microbiome and soil shifts to host performance, Spearman correlation analyses were conducted between the top 40 root-associated genera and soil properties or mulberry growth traits. The core genera associated with *P. koreensis* HLG18, including *Rhizorhapis*, *Bacillus*, *Caulobacter*, and *Rhodococcus*, displayed negative correlations with potassium, phosphorus, and iron concentrations (Fig. 6H). These taxa also showed strong positive correlations with mulberry growth parameters, including root and shoot biomass (Fig. 6I), suggesting a possible contribution to plant development. Overall, *P. koreensis* HLG18 inoculation was accompanied by shifts in endophytic community composition that coincided with changes in soil nutrient dynamics and improved host growth.

### *P. koreensis* HLG18 is associated with shifts in stress-mitigation metabolites and metabolic pathways in mulberry

**Fig 7:**
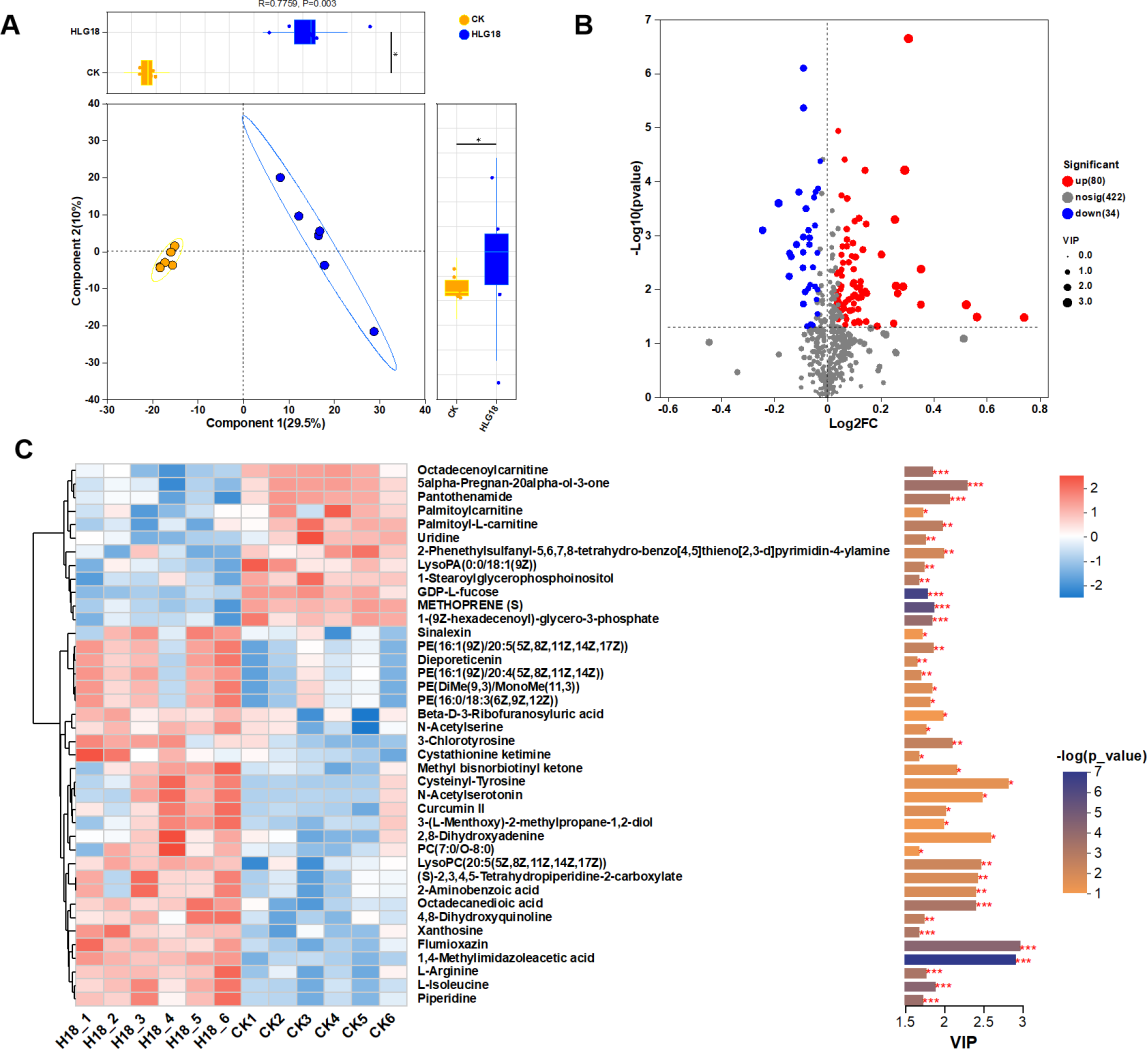
Impact of *P. koreensis* HLG18 strain on the root metabolome of mulberry. (**A**) PLS-DA analysis of root metabolite profiles. The ANOSIM R-value indicates the degree of separation between groups, with values closer to 1 indicating clearer intergroup differentiation. Corresponding *P* values reflect the statistical significance. (**B**) Volcano plots of differentially accumulated metabolites between *P. koreensis* HLG18-treated and control mulberry roots. The x-axis represents the log₂ fold change (log_2_FC), and the y-axis represents the −log_10_ (*P* value). (**C**) VIP scores of the top 40 differentially accumulated metabolites (DAMs) between control and *P. koreensis* HLG18-inoculated mulberry roots. Left: heatmap showing normalized relative abundance across samples. Right: bar plot of VIP scores. ***P < 0.001, **P < 0.01, *P < 0.05.

To assess the influence of *P. koreensis* HLG18 on mulberry root metabolism, untargeted metabolomics was performed. PLS-DA revealed clear separation between inoculated and control samples, indicating distinct metabolic profiles (Fig. 7A). KEGG annotation assigned metabolites to 15 secondary categories, with phospholipids as the most abundant group (Fig. S8A). These metabolites were primarily involved in ABC transporters, purine metabolism, and tryptophan metabolism (Fig. S8B). Consistently, Human Metabolome Database classification grouped metabolites into 11 superclasses and 20 classes, with glycerophospholipids prominently enriched (Fig. S9).

A total of 1,623 differentially accumulated metabolites (DAMs) were identified (*P* < 0.05; VIP > 1), including 987 detected in positive-ion mode and 636 in negative-ion mode (Table S6). Among these, 114 metabolites were annotated, with 80 upregulated and 34 downregulated in HLG18-treated roots (Fig. 7B). The most pronounced changes were observed in organoheterocyclic compounds (17 upregulated, 2 downregulated), lipids and lipid-like molecules (25 upregulated, 15 downregulated), and organic acids and derivatives (14 upregulated, 3 downregulated) (Table 2). KEGG enrichment analysis further indicated significant enrichment of pathways, including tryptophan metabolism, purine metabolism, and ABC transporters (Fig. S10). Notably, metabolites involved in tryptophan and purine pathways have been linked to plant growth promotion, antioxidant activity, and stress adaptation (43, 44).

**TABLE 2:** The classification of significantly different metabolites of mulberry root

Super class	Class	Number	Regulate
Organoheterocyclic compounds (13.19%)	Imidazopyrimidines	5	Up (4)/down (1)
	Indoles and derivatives	4	Up (4)
	Piperidines	3	Up (3)
	Tetrahydrofurans	1	Down (1)
	Pyridines and derivatives	1	Up (1)
	Azoles	1	Up (1)
	Furans	1	Up (1)
	Diazines	1	Up (1)
	Benzoxazines	1	Up (1)
	Thienoimidazolidines	1	Up (1)
Lipids and lipid-like molecules (27.78%)	Glycerophospholipids	20	Up (11)/down (9)
	Fatty acyls	12	Up (9)/down (3)
	Sphingolipids	3	Up (2)/down (1)
	Steroids and steroid derivatives	2	Down (2)
	Glycerolipids	2	Up (2)
	Prenol lipids	1	Up (1)
Organic acids and derivatives (11.81%)	Carboxylic acids and derivatives	17	Up (14)/down (3)
Benzenoids (3.47%)	Benzene and substituted derivatives	4	Up (4)
	Phenols	1	Up (1)
Nucleosides, nucleotides, and analogs (2.78%)	Pyrimidine nucleosides	1	Down (1)
	Pyrimidine nucleotides	1	Down (1)
	Purine nucleotides	1	Down (1)
	Purine nucleosides	1	Up (1)
Phenylpropanoids and polyketides (2.08%)	Flavonoids	2	Up (1)/down (1)
	Cinnamic acids and derivatives	1	Up (1)
Organic oxygen compounds (0.69%)	Organooxygen compounds	4	Up (3)/down (1)
Other (15.28%)	Other	22	Up (13)/down (9)

VIP-based ranking highlighted metabolites contributing most strongly to group discrimination, including 1,4-methylimidazoleacetic acid, flumioxazin, L-isoleucine, L-arginine, and piperidine (Fig. 7C). L-isoleucine, a precursor for jasmonoyl-isoleucine, is integral to jasmonate-mediated defense responses (45), whereas L-arginine contributes to redox regulation, stress signaling, and osmoprotection (46), consistent with their increased accumulation under HLG18 treatment.

To explore potential microbe-metabolite linkages, Pearson correlations were calculated between the top 40 DAMs and dominant endophytic genera (Fig. S11). Several genera, including *Rhizobium*, *Rhodococus*, *Bacillus*, *Caulobacter*, *Achromobacter*, and *Cellvibrio*, were strongly associated with DAM variation. Notably, genera (*Rhizorhapis*, *Bacillus*, *Caulobacter*, and *Rhodococcus*) were positively correlated with proline betaine, a well-established osmoprotectant implicated in abiotic stress tolerance (47). Overall, these findings suggest coordinated shifts in endophytic community composition and host metabolic adjustments following HLG18 inoculation.

## DISCUSSION

Waterlogging poses a major constraint to plant growth in fragile riparian ecosystems, such as those of the TGR, where it has markedly reduced mulberry vigor (1, 48). Harnessing beneficial endophytes as microbial inoculants has emerged as a sustainable strategy to enhance plant stress tolerance (49). Aligning with the well-documented role of *Pseudomonas* in plant stress resilience (50–53), our results reveal that *P. koreensis* HLG18 effectively promotes mulberry growth under both normal and waterlogged conditions. This effect mirrors that of *P. koreensis* S4T10, which ameliorates wheat tolerance to salinity and drought by stimulating antioxidant enzyme activities and photosynthetic pigments (54). Furthermore, the capacity of *Pseudomonas* species to dominate stress-adapted root microbiomes, as evidenced by their host-mediated enrichment in the soybean rhizosphere under salt stress (55), suggests that HLG18 may similarly establish a beneficial microbial niche in waterlogged environments. These findings highlight the potential of *P. koreensis* HLG18 as a bioinoculant for improving plant performance in the riparian zones of the TGR, China.

Inoculation with exogenous bacteria can reshape plant-associated microbiome across compartments, often by selectively enriching beneficial taxa that enhance plant health (19). For instance, the introduction of *Pseudomonas vancouverensis* M1 and *Enterobacter ludwigii* E15 altered the abundance of Enterobacteriaceae and increased rhizobacterial α-diversity in *Vallisneria natans* (56). Here, *P. koreensis* HLG18 similarly elicited distinct compositional shifts in the endophytic bacterial communities in mulberry. Notably, several root-associated genera implicated in plant growth promotion and abiotic stress tolerance, such as *Rhizorhapis*, *Bacillus*, *Caulobacter*, and *Rhodococcus*, were significantly elevated in inoculated plants (57–60). Importantly, the increased abundances of these genera were correlated positively with mulberry growth parameters. This correlation suggests that the growth-promoting effects of *P. koreensis* HLG18 may be partially mediated by modulation of the host endophytic microbiome to favor these beneficial taxa. Nevertheless, further isolation and functional characterization of these genera are required to fully elucidate their contributions to plant growth.

Plant growth promotion by microbes arises from dynamic exchanges of metabolites, energy, and signals among microbial communities, plant roots, and the soil environment (60, 61). Inoculation of *P. koreensis* HLG18 induced measurable shifts in soil nutrient profile, implicating a plant-microbe feedback loop capable of modifying edaphic conditions. This phenomenon is also documented for the endophyte *Pseudomonas* sp. E3, which alters nitrogen and phosphorus availability in *Solanum nigrum* (62). The ability of HLG18 to produce siderophores and solubilize minerals likely underpins this process, thereby facilitating nutrient acquisition by the mulberry host. Moreover, the negative correlation observed between enriched bacterial taxa and soil nutrient levels suggests that nutrient mobilization emerges from the synergistic activities of HLG18 and the restructured microbial consortia.

Plant-endophyte mutualism enhances stress resilience by facilitating the accumulation of protective metabolites (63). For example, *Lactobacillus parafarraginis* stimulates amino acid metabolism in hybrid *Pennisetum* (64), while *Bacillus amyloliquefaciens* EZ99 alters sucrose and mannobiose metabolism in potato (65). Inoculation of HLG18 also triggered significant alterations in the root metabolome, particularly in purine metabolism, a central pathway for stress adaptation (66). Given that the purine derivative xanthine enhances *Pseudomonas* colonization and soybean salt tolerance (55), a comparable mechanism may underlie HLG18-mediated waterlogging resilience. Simultaneously, we observed substantial increases in tryptophan and its derivatives and L-arginine, which are established regulators of plant development and stress responses (46, 67, 68). Moreover, enrichment of specific bacterial taxa following inoculation was positively correlated with levels of the osmoprotectant proline betaine (47, 69), suggesting a potential feedback loop whereby HLG18-induced metabolic changes modulate the root microbiome.

### Conclusion

**Fig 8:**
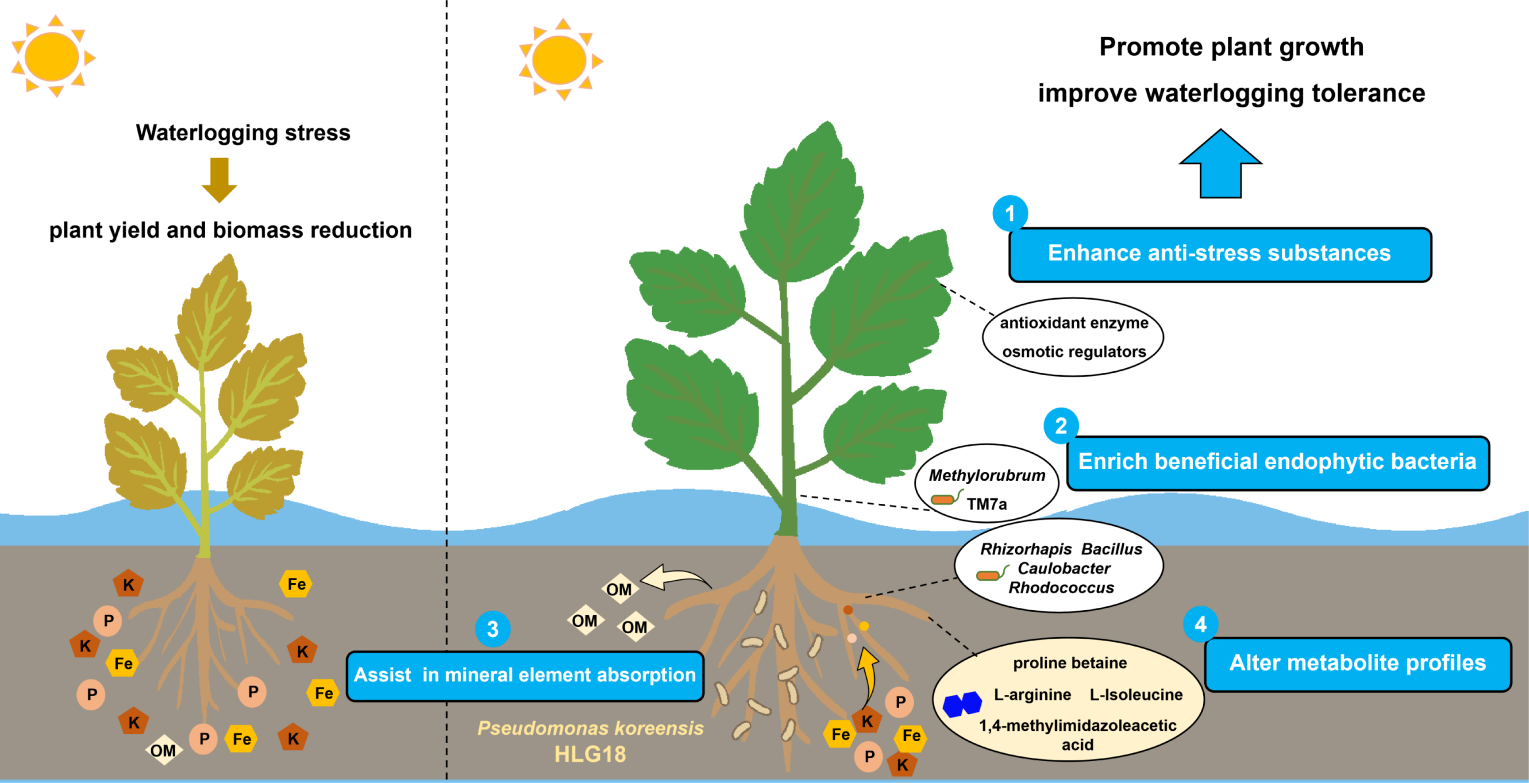
Proposed model for the mechanisms by which *P. koreensis* HLG18 enhances waterlogging tolerance in mulberry. Under waterlogging conditions, *P. koreensis* HLG18 increased mulberry antioxidant enzyme activities and osmoprotectant accumulation. Inoculation with HLG18 also elevated the relative abundances of keystone endophytic bacteria, such as *Rhizorhapis*, *Bacillus*, *Caulobacter*, and *Rhodococcus* in roots, while decreasing soil concentrations of iron (Fe), potassium (K), and phosphorus (P), and increasing organic carbon and organic matter (OM). In addition, *P. koreensis* HLG18 inoculation was associated with changes in the root metabolite profile, with the accumulation of defensive metabolites (e.g., L-arginine, proline betaine). Together, these coordinated variations in the microenvironment and root metabolome were consistently observed in mulberry exhibiting enhanced growth performance and waterlogging tolerance.

In summary, this study demonstrates that the endophyte *P. koreensis* HLG18 significantly enhances mulberry tolerance to waterlogging stress. This improvement is mainly associated with strengthened antioxidant capacity and increased accumulation of stress-mitigation metabolites, accompanied by coordinated variations in the endophytic community, soil physicochemical properties, and host root metabolism (Fig. 8). These findings highlight the potential of endophyte-assisted strategies to enhance plant waterlogging tolerance and support vegetation restoration in the riparian zone of the TGR. Moreover, it provides a reference for the exploration of beneficial microbial resources in sustainable agriculture.

## Data Availability

The 16S rRNA gene sequence of *Pseudomonas koreensis* HLG18 has been deposited in GenBank under accession number ON103349. The complete genome sequence of *Pseudomonas koreensis* HLG18 has been deposited in GenBank under BioSample accession number SAMN49959647 and BioProject accession number PRJNA1291291. The raw Illumina sequence data were submitted to the NCBI Sequence Read Archive (SRA) database under BioProject accession number PRJNA1291370.

## SUPPLEMENTAL MATERIAL

Supplemental material spectrum.02959-25-s0001.docx Tables S1 to S6; Fig. S1 to S11.

ASM does not own the copyrights to Supplemental Material that may be linked to, or accessed through, an article. The authors have granted ASM a non-exclusive, world-wide license to publish the Supplemental Material files. Please contact the corresponding author directly for reuse.
