# Supplementary material for: Pseudomonas koreensis HLG18 improves mulberry waterlogging resilience in riparian zone by synergistically modulating endophytic microbiome and metabolic profiles
Source: Microbiol Spectr. 2026 Feb 9;14(3):e02959-25. doi: 10.1128/spectrum.02959-25 (PMC12955400; doi:10.1128/spectrum.02959-25)
Supplement: Supplemental material — Tables S1 to S6; Fig. S1 to S11. [file spectrum.02959-25-s0001.docx]

***Pseudomonas koreensis* HLG18 improves mulberry waterlogging resilience in riparian zone by synergistic modulating endophytic microbiome and metabolic profiles**

Ting Ou^1^, Haiying Gao^1^, Yuping Xiong^1^, Kun Jiang^1^, Changyu Qiu^2^, Kai Lin^1^, Xiaojiao Liu^1^, and Jie Xie^1#^

^1^ State Key Laboratory of Resource Insects, College of Sericulture, Textile and Biomass Science, Southwest University, Chongqing 400715, China.

^2^ Guangxi Key Laboratory of Sericultural Genetic Improvement and Efficient Breeding, Guangxi Zhuang Autonomous Region Sericultural Technology Promotion Station, Nanning, Guangxi Zhuang Autonomous Region 530007, China.

^#^Address correspondence to Jie Xie. Email address: [healthjie@swu.edu.cn](mailto:healthjie@swu.edu.cn)

**Table S1** The mulberry growth parameters of Spearman correlation analysis

| Treatment | SL (cm) | SW (mg) | RL (cm) | RW (mg) | SA (cm^2^) | LR |
| --- | --- | --- | --- | --- | --- | --- |
| CK | 12.22±0.52 | 1.08±0.20 | 6.64±1.34 | 0.11±0.05 | 1483.14±1135.34 | 370.13±241.49 |
| *P. koreensis* HLG18 | 14.86±1.19 | 1.10±0.21 | 9.71±1.82 | 0.39±0.13 | 8301.16±1922.77 | 822.50±66.48 |

SL and RL represent mulberry shoot length and main root length, respectively. SW and RW represent the dry weight of mulberry root and shoot, respectively. SA and LR represent the root shadow area and root tip number, respectively.

**Table S2** Screening of potential plant growth-promoting endophytic bacteria isolated from mulberry in the riparian zone

| Isolates | Genus | ^a^IAA-production | ^b^P-solubilization |
| --- | --- | --- | --- |
| HNG24 | *Pantoea* sp. | 1 | 1 |
| HNG25 | *Acinetobacter* sp. | 1 | 2 |
| HLJ16 | *Curtobacterium* sp. | 1 | 1 |
| HLJ20 | *Atlantibacter* sp. | 1 | 1 |
| HLJ4 | *Lelliottia* sp. | 1 | 2 |
| HLG18 | *Pseudomonas* sp. | 3 | 3 |

^a^IAA production: scored as ‘1’ for weak red coloration and ‘3’ for strong red coloration.

^b^P-solubilization: scored based on the ratio of halo diameter to colony diameter (HD/CD). ‘1’ indicates a weak halo (1 ≤ HD/CD < 2), ‘2’ indicates a clear halo (2 ≤ HD/CD < 3), and ‘3’ indicates a strong halo (3 ≤ HD/CD < 4).

**Table S3** Physiological and biochemical characteristics of the HLG18 strain

| Items | Results | Items | Results |
| --- | --- | --- | --- |
| Glucose | + | Semi-solid agar | + |
| Xylose | + | H_2_S | − |
| Maltose | + | Nitrate | − |
| Peptone | − | Mannitol | − |
| Arginine | + | Citrate | + |

‘+’ represents positive (growth or reaction) and ‘–’ represents negative (no growth or no reaction).

**Table S4** Genomic feature comparison of *P. koreensis* HLG18 and representative *Pseudomonas koreensis* strains

| Features | *Pseudomonas koreensis* HLG18 | *Pseudomonas koreensis* BS3658 | *Pseudomonas koreensis* D26 | *Pseudomonas koreensis* CRS05-R5 |
| --- | --- | --- | --- | --- |
| Genome size (Mb) | 6.80 | 6.12 | 6.30 | 5.99 |
| G+C content (%) | 60.43 | 60.50 | 59.60 | 60.60 |
| tRNA number | 74 | 73 | 69 | 73 |
| rRNA number | 20 | 19 | 16 | 19 |
| Total predicted CDS | 5,644 | 5,497 | 5,592 | 5,295 |
| Plasmid number | – | – | – | – |
| GenBank accession | CP195831 | GCA_900101415 | GCA_001605965 | GCA_001654515 |

‘–’ represents none of plasmids in strains and CDS represents protein-coding sequences.

**Table S5** Identification of gene clusters potentially involved in secondary metabolite synthesis in the *P. koreensis* HLG18 genome

| Cluster | Cluster Type | From | To | Most similar known cluster | MIBiG accession |
| --- | --- | --- | --- | --- | --- |
| 1 | NRPS | 915,744 | 1,001,012 | Pf-5 pyoverdine | BGC0000413 |
| 2 | NRPS | 1,355,084 | 1,421,974 | Lokisin | BGC0001980 |
| 3 | NRPS | 1,450,929 | 1,510,006 | Chitinimide | BGC0002503 |
| 4 | Ranthipeptide | 3,133,497 | 3,154,927 | Pf-5 pyoverdine | BGC0000413 |
| 5 | NRPS | 3,185,699 | 3,238,700 | Pf-5 pyoverdine | BGC0000413 |
| 6 | NAGGN | 3,383,261 | 3,398,034 | − | − |
| 7 | Redox-cofactor | 4,550,977 | 4,573,145 | Lankacidin C | BGC0001100 |
| 8 | Arylpolyene | 5,611,718 | 5,655,331 | APE Vf | BGC0000837 |

NRPS and NAGGN represent non-ribosomal peptide synthetase and N-acetylglutaminylglutamine amide, respectively.

**Table S6** Number of differentially accumulated metabolites between *P. koreensis* HLG18-treated and control mulberry roots

| Modes | Total metabolites | Known metabolites | Unknown metabolites |
| --- | --- | --- | --- |
| Positive mode | 987 | 69 | 918 |
| Negative mode | 636 | 45 | 591 |


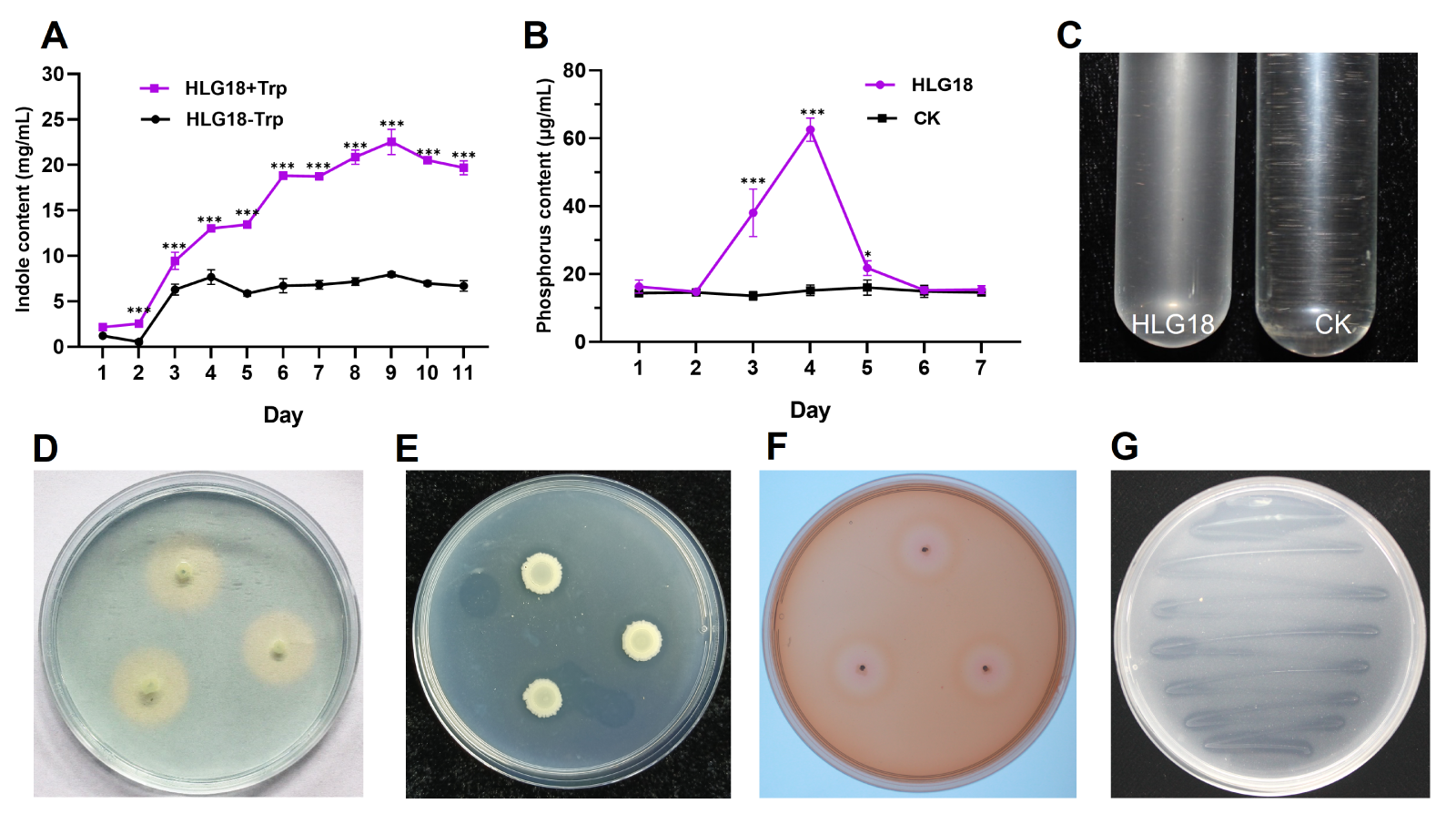


**FIG S1** Characterization of *P. koreensis* HLG18 strain for plant growth-promoting traits and extracellular enzymes. (A) Indole production in LB medium with or without 0.1% L-tryptophan (Trp) supplementation, measured daily over an 11-day incubation at 28˚C. (B) Phosphorus solubilization in Pikovskaya’s liquid medium over a 7-day incubation at 28˚C. CK, Pikovskaya’s liquid medium without strain. (C) ACC deaminase activity measured on ADF medium after 2 d at 28˚C. (D) Siderophore production evaluated using chrome azurol S agar after 3 d at 28˚C. (E) Potassium solubilization evaluated on modified Aleksandrov agar supplemented with potassium feldspar powder after 3 d at 28˚C. (F) Cellulase activity tested on carboxymethyl cellulose medium after 3 d at 28˚C. (G) Nitrogen fixation ability determined on Ashby’s medium after 3 d at 28˚C. Data in (A) and (B) represent mean ± SEM (*n* = 4). Significant difference was determined by the two-tailed Student’s t-test: **p* < 0.05, ****p* < 0.001.

**
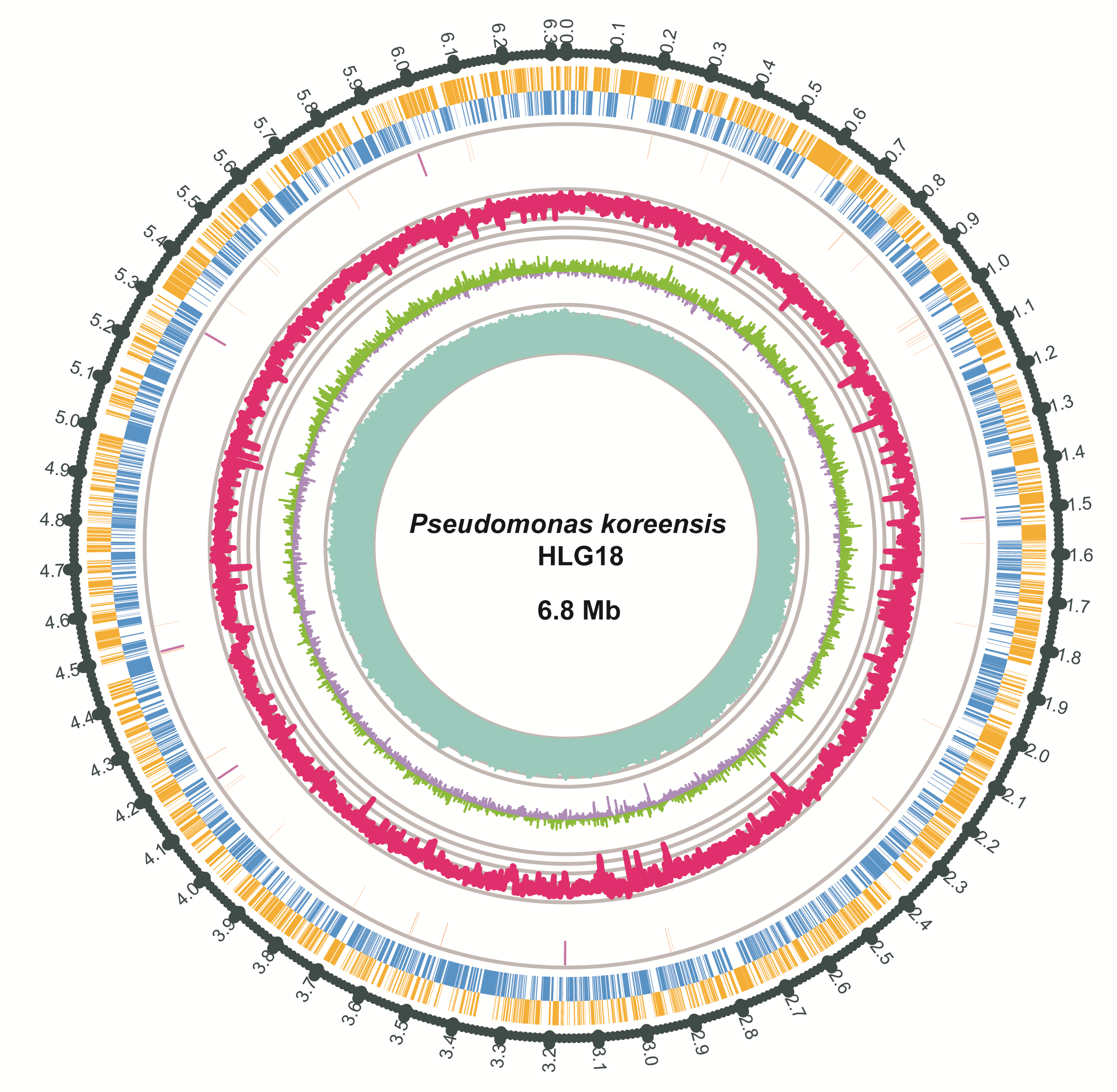
**

**FIG S2** Circular genome map of *P. koreensis* HLG18 strain. The concentric rings from outermost to innermost represent: (1) genome size (scale in Mb), (2) predicted coding sequences on the forward (sense) strand, (3) predicted coding sequences on the reverse (antisense) strand, (4) annotated non-coding RNAs (ncRNAs), (5) GC content, (6) GC skew, and (7) sequencing depth.


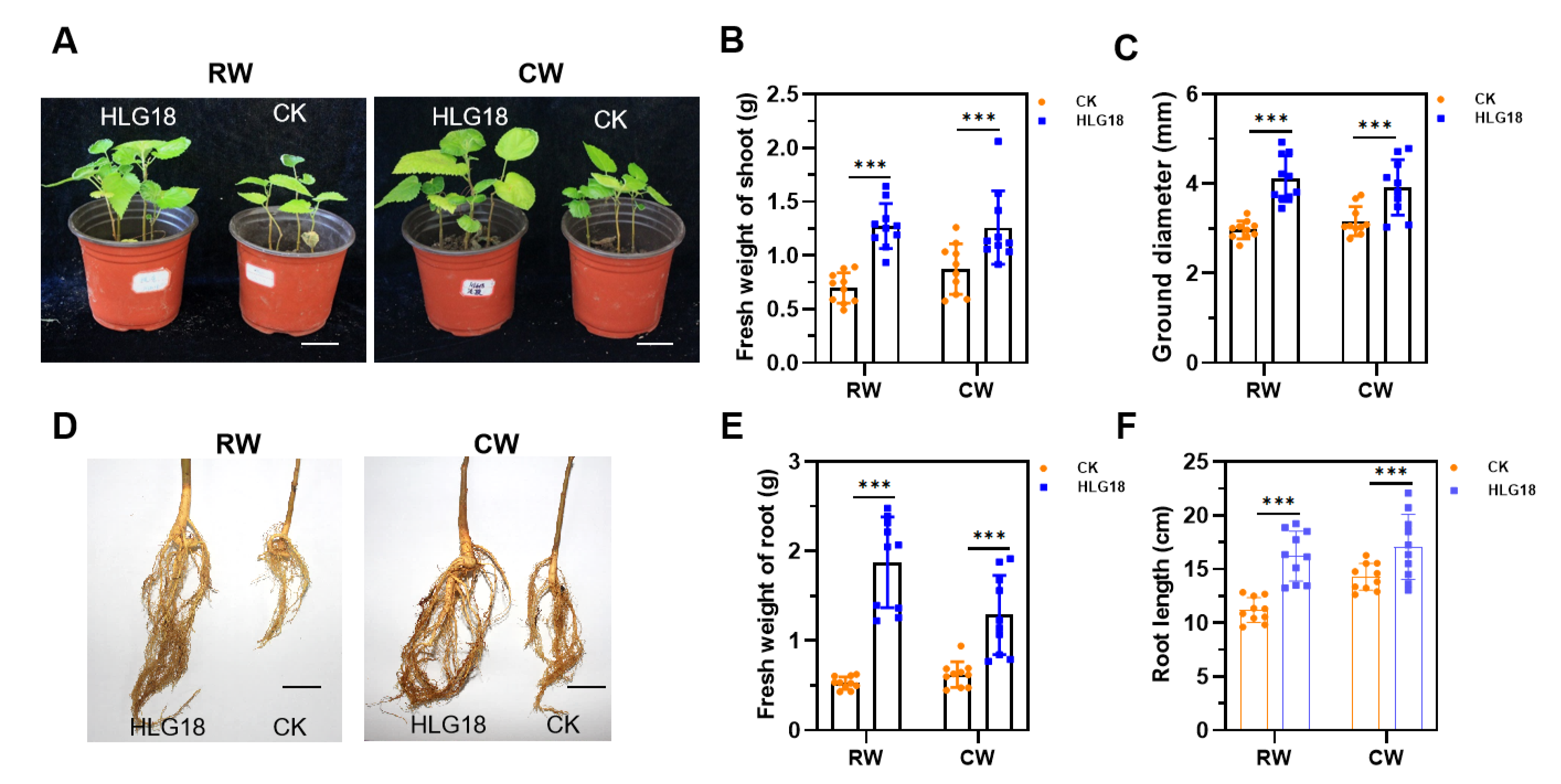


**FIG S3** *P. koreensis* HLG18 promotes mulberry recovery after waterlogging stress. (A) Representative images of aboveground mulberry phenotype following recovery. Scale bars = 3 cm. (B) Shoot fresh weight. (C) Ground diameter. (D) Representative images of mulberry root architecture post-recovery. (E) Root fresh weight. (F) Root length. Data represent mean ± standard deviation (*n* = 10). Significant differences were determined by one-way ANOVA followed by Tukey’s tests. *** *p* < 0.001. RW, root waterlogging; CW, complete waterlogging.


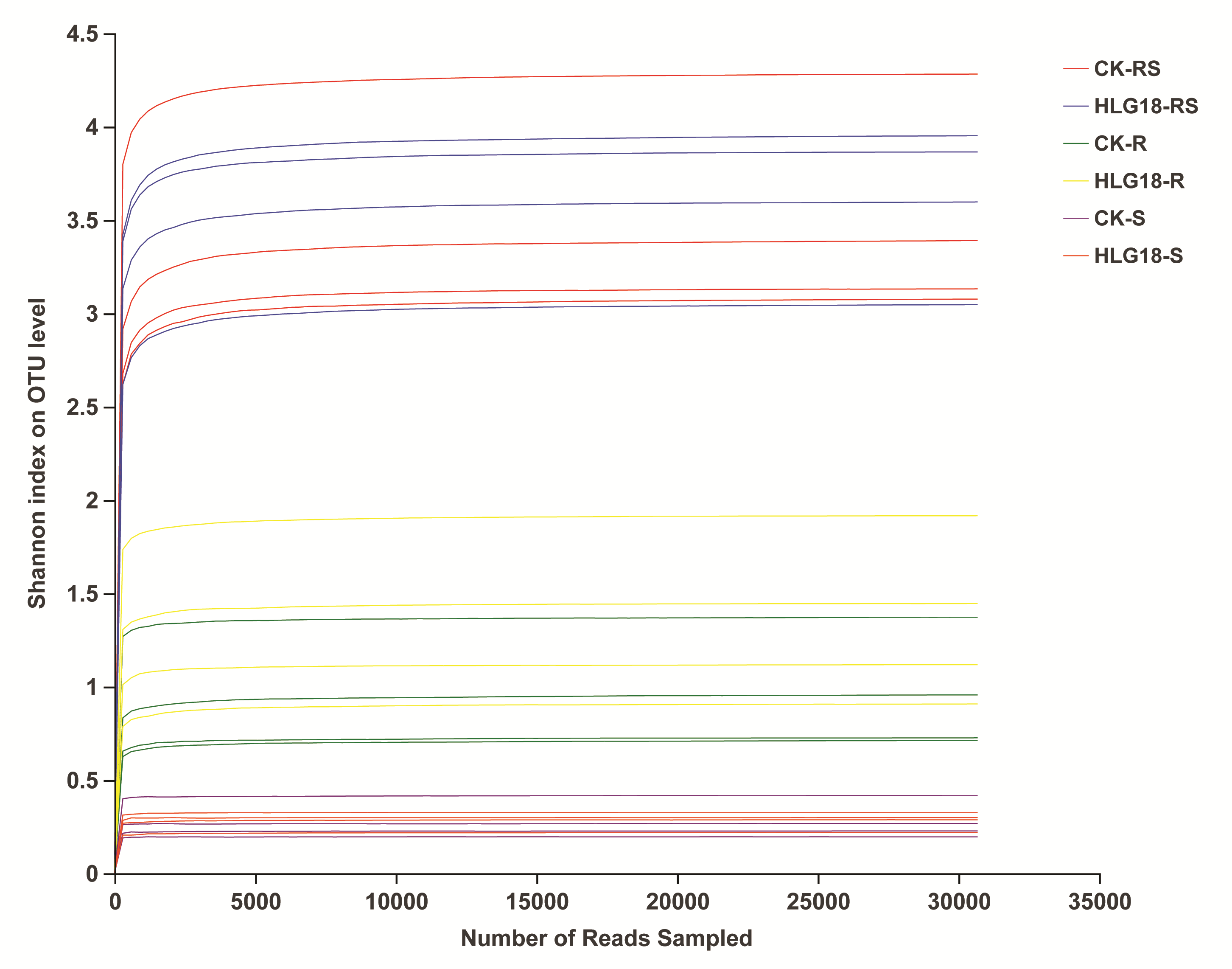


**FIG S4** Rarefaction curves of bacterial communities across different mulberry sample types. Rarefaction curves based on observed OTUs (97% sequence similarity) indicate bacterial richness in various compartments. The x-axis represents the number of sequencing reads per sample, and the y-axis shows the number of observed OTUs. CK-RS, CK-R, and CK-S represent bacterial communities from rhizosphere soil, root, and stem in water control groups, respectively. HLG18-RS, HLG18-R, and HLG18-S represent bacterial communities from rhizosphere soil, root, and stem in *P. koreensis* HLG18-inoculated group, respectively.


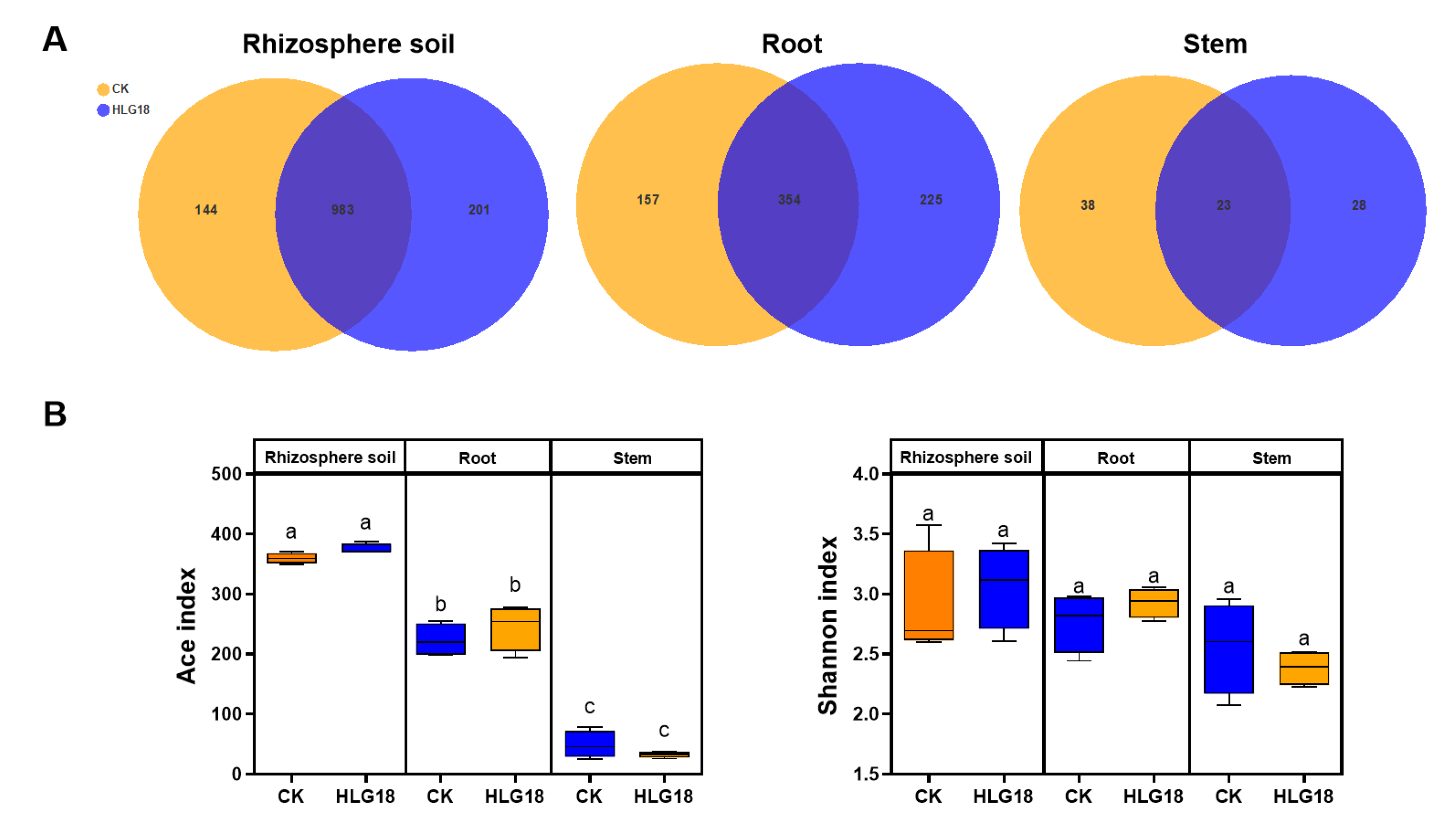


**FIG S5** Venn diagram and α-diversity analysis of bacterial community across different sample types. (A) Venn diagram showing the number of shared and unique OTUs among different groups. The values represent the number of OTUs in each group. (B) α-diversity indices of the bacterial community. Richness is assessed using the Ace index (left), and diversity is evaluated using the Shannon index (right). Bars with different letters indicate statistically significant differences based on one-way ANOVA followed by Tukey’s test (*p* < 0.05). Data represent mean ± standard deviation (*n* = 4).


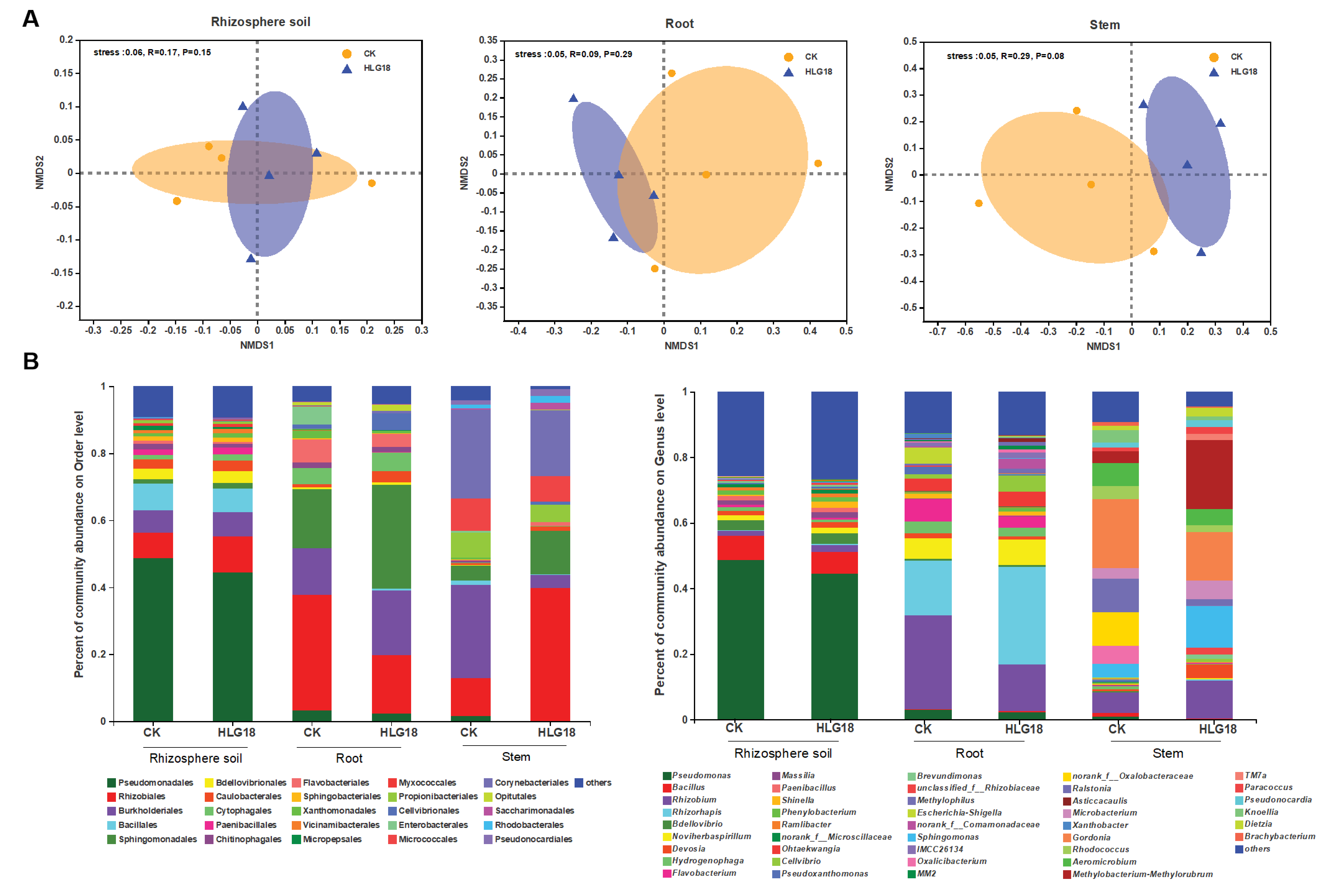


**FIG S6** Effect of *P. koreensis* HLG18 on mulberry-associated bacterial communities. (A) Non-metric multidimensional scaling analysis based on Bray-Curtis distances, illustrating the β-diversity of bacterial communities in mulberry rhizosphere soil, roots, and stems. (B) Taxonomic composition of bacterial communities at the order level (left) and genus level (right) in the rhizosphere soil, root, and stem. Taxa with a relative abundance < 1% were g classified as “others.” Each bar represented the average of four biological replicates.


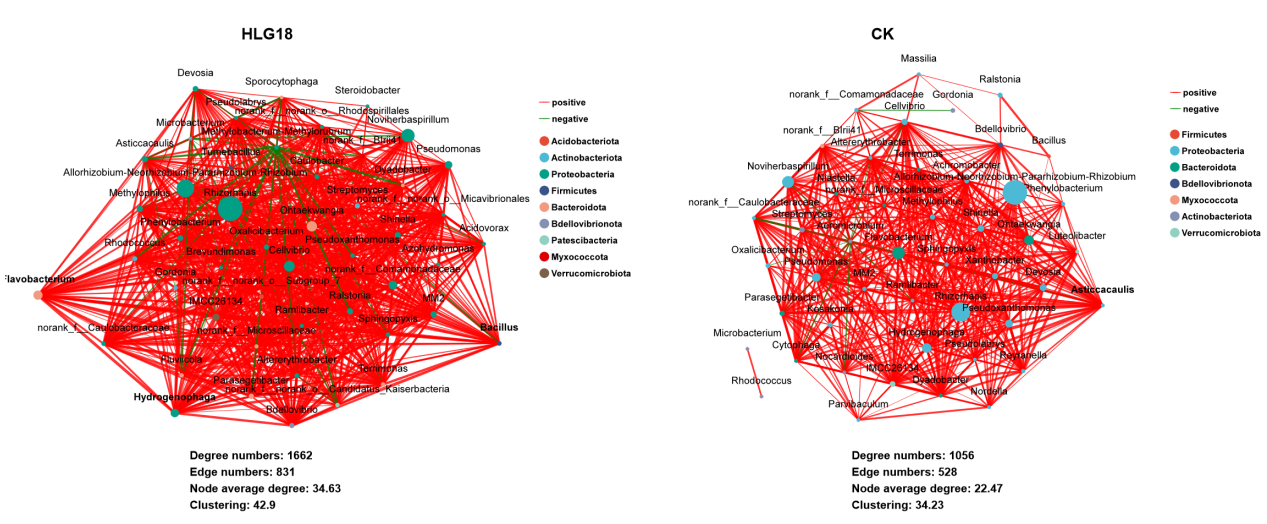


**FIG S7** Correlation network analysis of endophytic bacterial communities in mulberry root. Nodes represent bacterial genera and node size indicates their relative abundance. Edge colors represent the type of correlation between taxa: red indicates positive correlations and green indicates negative correlations. Edge thickness reflects the strength of the correlation coefficient, with thicker edges representing stronger associations.


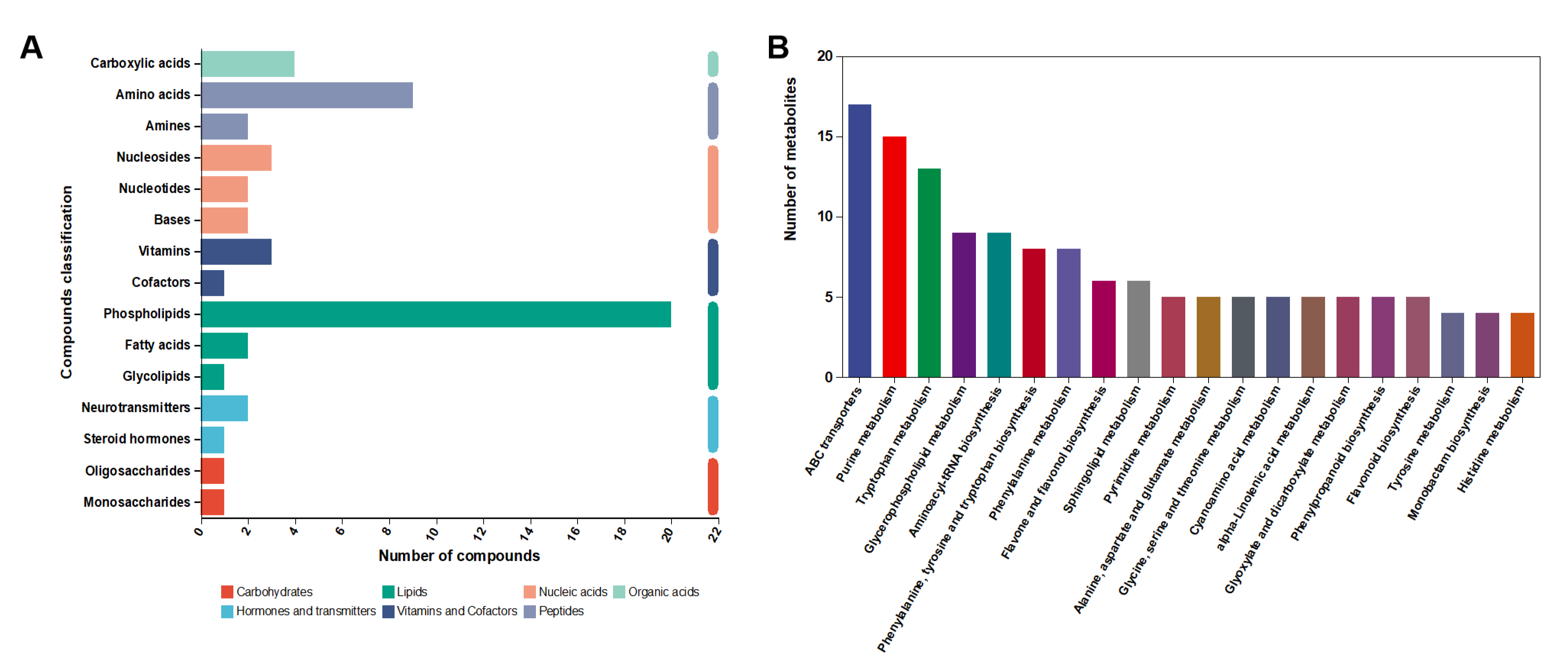


**FIG S8** Classification and pathway enrichment analysis of mulberry root metabolites based on the KEGG database. (A) Metabolites classification. The y-axis represents KEGG compound categories (second-level classification), and the x-axis indicates the number of metabolites annotated in each category. Bar colors represent first-level KEGG classification groups. (B) Pathway enrichment of annotated metabolites. The y-axis shows the KEGG secondary-level metabolic pathways, and the x-axis indicates the number of metabolites associated with each pathway.


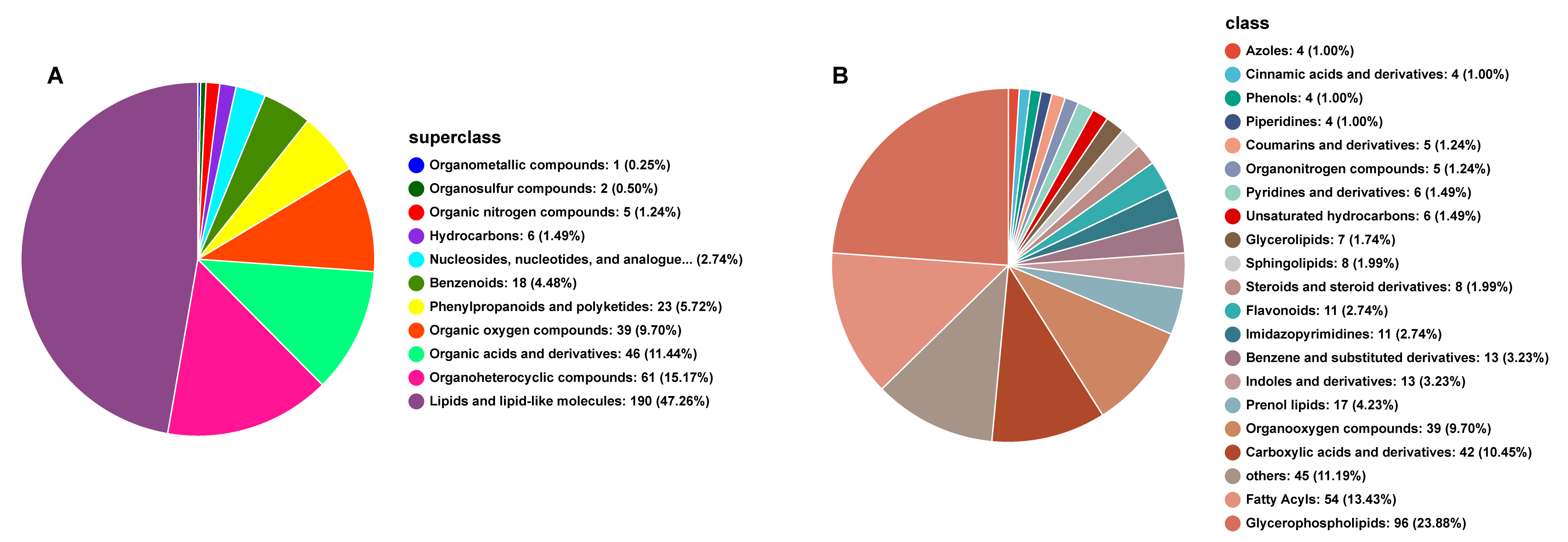


**FIG S9** Classification of mulberry root metabolites based on the HMDB database. (A) Superclass-level classification. (B) Class-level classification.


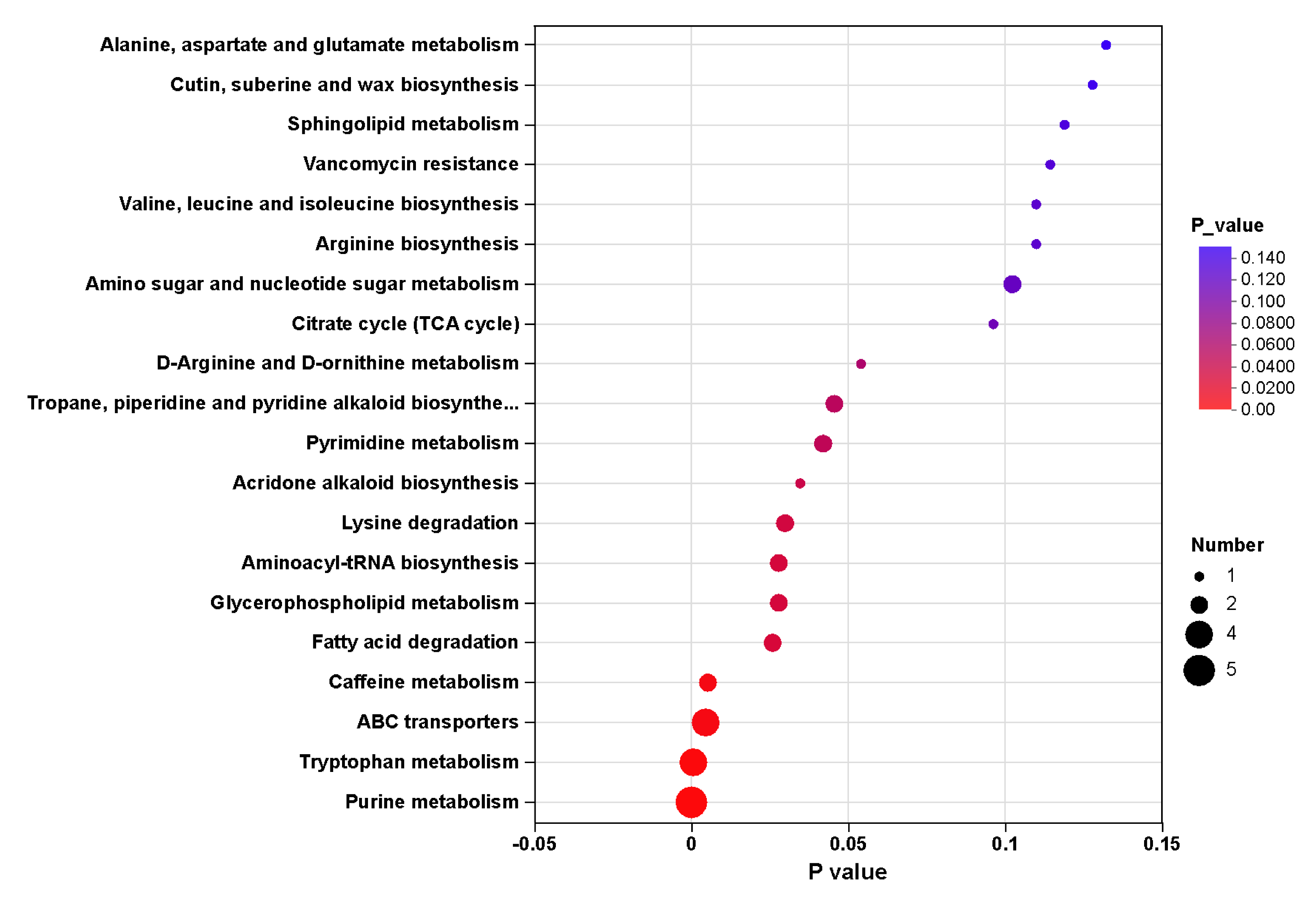


**FIG S10** KEGG pathway enrichment analysis of differentially accumulated metabolites in mulberry roots. The x-axis indicates the enrichment ratio, and the y-axis displays the KEGG pathways significantly enriched among the differentially accumulated metabolites. Bubble size indicates the number of metabolites enriched in each pathway, and bubble color represents the *p*-value, reflecting the statistical significance of enrichment.


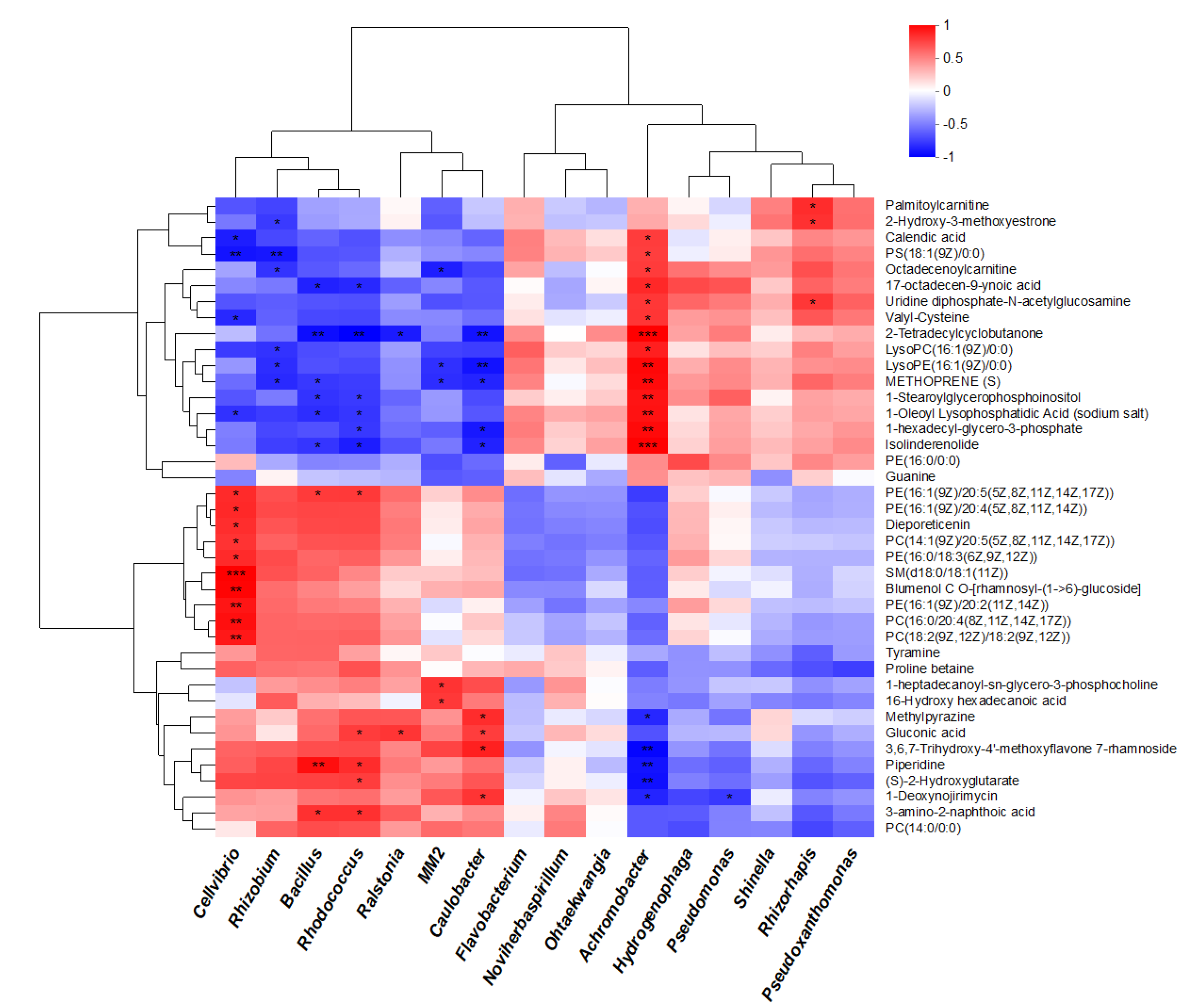


**FIG S11** Correlation analysis between the top 40 differentially accumulated metabolites and bacterial genera in mulberry roots. Rows represent individual metabolites, and columns represent bacterial genera. Each square displays the Pearman correlation coefficient between a metabolite and bacterial genus, with color intensity indicating the strength and direction of the correlation. Asterisks indicate statistical significance: ****p* < 0.001, ** *p* < 0.01, * *p* < 0.05.
